# Dengue in the Middle East and North Africa: A Systematic Review

**DOI:** 10.1371/journal.pntd.0005194

**Published:** 2016-12-07

**Authors:** John M. Humphrey, Natalie B. Cleton, Chantal B. E. M. Reusken, Marshall J. Glesby, Marion P. G. Koopmans, Laith J. Abu-Raddad

**Affiliations:** 1 Division of Infectious Diseases, Department of Medicine, Weill Cornell Medical College, New York, New York, United States of America; 2 Erasmus Medical Centre, Rotterdam, The Netherlands; 3 National Institute for Public Health and Environment (RIVM), Bilthoven, The Netherlands; 4 Department of Healthcare Policy and Research, Weill Cornell Medical College, Cornell University, New York, New York, United States of America; 5 Infectious Disease Epidemiology Group, Weill Cornell Medical College in Qatar, Cornell University, Qatar Foundation, Education City, Doha, Qatar; 6 College of Public Health, Hamad bin Khalifa University, Qatar Foundation, Education City, Doha, Qatar; University of Heidelberg, GERMANY

## Abstract

**Background:**

Dengue virus (DENV) infection is widespread and its disease burden has increased in past decades. However, little is known about the epidemiology of dengue in the Middle East and North Africa (MENA).

**Methodology / Principal Findings:**

Following Cochrane Collaboration guidelines and reporting our findings following PRISMA guidelines, we systematically reviewed available records across MENA describing dengue occurrence in humans (prevalence studies, incidence studies, and outbreak reports), occurrence of suitable vectors (*Aedes aegypti* and *Aedes albopictus)*, and DENV vector infection rates. We identified 105 human prevalence measures in 13 of 24 MENA countries; 81 outbreaks reported from 9 countries from 1941–2015; and reports of *Ae*. *aegypti* and/or *Ae*. *albopictus* occurrence in 15 countries. The majority of seroprevalence studies were reported from the Red Sea region and Pakistan, with multiple studies indicating >20% DENV seroprevalence in general populations (median 25%, range 0–62%) in these subregions. Fifty percent of these studies were conducted prior to 1990. Multiple studies utilized assays susceptible to serologic cross-reactions and 5% of seroprevalence studies utilized viral neutralization testing. There was considerable heterogeneity in study design and outbreak reporting, as well as variability in subregional study coverage, study populations, and laboratory methods used for diagnosis.

**Conclusions / Significance:**

DENV seroprevalence in the MENA is high among some populations in the Red Sea region and Pakistan, while recent outbreaks in these subregions suggest increasing incidence of DENV which may be driven by a variety of ecologic and social factors. However, there is insufficient study coverage to draw conclusions about *Aedes* or DENV presence in multiple MENA countries. These findings illustrate the epidemiology of DENV in the MENA while revealing priorities for DENV surveillance and *Aedes* control.

## Author Summary

Dengue is a mosquito-transmitted flavivirus whose global distribution and disease incidence has increased in recent decades. In the Middle East and North Africa, the epidemiology of dengue remains poorly characterized despite increasing reports of outbreaks and transmission in new areas. In order to understand the evidence supporting the epidemiology of this virus in the region and the areas in need of further research, we conducted a systematic review of studies reporting human prevalence, incidence, and infection rates in the virus’ main mosquito vectors, *Aedes aegypti* and *Aedes albopictus*. Among the studies identified, the Red Sea subregion and Pakistan reported the highest seroprevalence estimates for dengue. However, we encountered substantial heterogeneity in the distribution, quality, and quantity of published studies. These findings inform future research and surveillance priorities for DENV in the MENA region.

## Introduction

Dengue virus (DENV) is a globally distributed flavivirus with nearly 400 million estimated annual infections and a growing geographic distribution and disease burden [1–3]. DENV has a historic presence in the Middle East and North Africa (MENA), with outbreaks of dengue and dengue-like disease reported across much of the Eastern Mediterranean region in the 19^th^ and early 20^th^ centuries [4, 5]. Today, DENV may be resurging in the MENA [6, 7], with recent outbreaks of unprecedented or previously unrecognized magnitude occurring in the Arabian Peninsula and Pakistan [8, 9], and a 2015 outbreak in Egypt that occurred following a decades-long absence of reported cases from that country [10]. Still, despite increasing global concern about the threat of *Aedes*-transmitted arboviruses, the epidemiology of DENV in the MENA region remains largely uncharacterized.

Understanding the epidemiology of DENV in the MENA represents an ongoing challenge for multiple reasons [11]. Inadequate human and vector surveillance, non-reporting of illness syndromes, and poor diagnostic capacity limit DENV detection in many countries, resulting in delays in outbreak recognition and sparse data with which to estimate disease burden and infection rates [12–14]. Case series, outbreak reports, and national notification reports, which contribute much to the epidemiologic knowledge of DENV, may also contain bias in reflecting only those areas with sufficient capacity to detect and report DENV when it occurs [1]. Moreover, clinical diagnosis of DENV infection in the absence of laboratory confirmation is often unreliable [12, 15–18]. Cross-sectional serologic surveys for DENV exposure have the potential to shed light on the broader population burden of DENV without these biases. However, serologic cross-reactions among antibody-based assays for flaviviruses can limit the reliability of such studies in the absence of confirmatory testing, though the latter is difficult to perform and often unavailable [19, 20].

To further the knowledge of the epidemiology of DENV in the MENA, we undertook a comprehensive summary and appraisal of published DENV prevalence, incidence, vector infection rates, reported outbreaks, and *Aedes* occurrence reports in the MENA region. This report aims to enhance the understanding of the epidemiology of DENV in the MENA while informing priorities for future research.

## Materials and Methods

### Objectives

The objective of this study was to characterize the epidemiology of DENV in the MENA region through a systematic review of human prevalence and incidence studies and infection rates in *Aedes* mosquitoes. We also aimed to summarize reported human outbreaks and *Ae*. *aegypti and Ae*. *albopictus* occurrence in the region. The original search was last updated on December 9, 2015.

### Eligibility criteria

Table 1 displays the eligibility criteria. In brief, studies containing primary prevalence, incidence, and vector infection rates for DENV in the MENA region were considered eligible for the systematic review. Publication year was not considered an inclusion criterion, as we reasoned that the historic distribution of DENV could be useful in understanding its current epidemiology by depicting ecologically viable regions in which DENV transmission continues to occur or could re-emerge. For incidence studies, those that reported the number of acute infections or seroconversions over any time interval were eligible. Vector infection rate studies were included if they contained a measure of the estimated proportion of infected *Ae*. *aegypti* or *Ae*. *albopictus* at a given time and setting in the MENA region.

**Table 1:** Criteria for study inclusion or exclusion.

**Study type**	**Inclusion Criteria**	**Exclusion Criteria**
Human prevalence/incidence		
publication characteristics	Full article or abstract published in any year, language, setting, or population in the MENA region; any seroconversion interval for incidence studies	Case reports, case series, editorials, letters to editors, reviews, commentaries, qualitative studies, basic science research studies, studies from countries outside the MENA region
study design	Any randomized or non-randomized design	Non-empirical research/modelled data
outcomes	DENV seroprevalence or prevalence of laboratory-confirmed infection; DENV incidence (by any laboratory method)	No human prevalence or incidence measure reported
Vector infection rate	Reported *Ae*. *aegypti* or *Ae*. *albopictus* infection rates by any laboratory method	Basic science research studies, infection rates in other mosquito species or non-MENA country

### Outcomes

For the systematic review, the primary outcomes were DENV human prevalence, incidence, and vector infection rates in the MENA region. Secondary outcomes were reports of dengue outbreaks and vector occurrence.

### Data sources and search strategy

We conducted a systematic search for DENV in the MENA following Cochrane Collaboration guidelines [21] and reported our findings using the Preferred Reporting Items for Systematic reviews and Meta-analyses (PRISMA) guidelines [22]. The PRISMA checklist is found in S1 Fig and our search criteria in S2 Fig. Briefly, we searched PubMed, Embase, the World Health Organization (WHO) Index Medicus for the Eastern Mediterranean Region and WHO African Index Medicus without publication date or language restrictions, using text and MeSH/Emtree terms exploded to include all subheadings. Our review covered the 23 countries included in the MENA definitions of the WHO/EMRO, World Bank, and the Joint United Nations Programme on HIV/AIDS (UNAIDS) for consistency with earlier regional analyses of various infectious diseases including HIV [23].

### Study selection

For each search, titles and abstracts were imported into Endnote (Thompson Reuters, Philadelphia, PA, USA), duplicates were removed, and were screened by one author (JH) with potential eligibility determined by consensus with a second author (NC) when eligibility was unclear. Full texts of potentially relevant records were retrieved and assessed for eligibility, contacting the author of the report as necessary. Reference lists of all potentially eligible articles and reviews were also searched. In this study, ‘report’ refers to the document (paper, abstract, or public health record) containing an outcome measure of interest, while ‘study’ refers to the outcome measure(s) within that report. Hence, reports could contribute more than one study, though multiple reports of the same study were counted only once.

### Data Extraction and Synthesis

Data were extracted by one of the authors (JH) using a pre-piloted data extraction form and entered into a database created in Microsoft Access. Data from reports in English were extracted from the full texts, while reports in French (n = 6), Turkish (n = 3), Dutch (n = 1), and German (n = 1) were extracted from the abstracts and full texts with the help of online language software and French, Turkish, and German language speakers [24]. There were no records in other languages. Studies were compiled by country and organized by year, using separate tables for human prevalence, incidence, and vector infection rates. Prevalence studies were further stratified as follows: 1) *general prevalence studies* measuring the prevalence of anti-DENV antibodies among populations without acute infection (e.g. DENV exposure); and 2) *acute DENV infection studies* assessing the prevalence of laboratory-confirmed DENV infection in those with a) undifferentiated acute febrile illness (AFI) and b) suspected DENV infection (Table 2). These stratifications were made because of the different study aims and probabilities of having laboratory evidence of DENV infection in each of these populations. Finally, the geographic distribution of all included prevalence studies were mapped according to the first-level administrative division (e.g. state, province) in which each study was conducted (Tableau Software, Seattle, WA, USA).

**Table 2:** Definitions of human prevalence study populations identified through the systematic review.

**Study Population**	**Definition**
General prevalence	Seroprevalence studies reporting anti-DENV IgG prevalence measures among individuals not suspected to have acute dengue infection, including community members, blood donors, military, students, and hospitalized patients and outpatients receiving care for non-febrile illnesses.
Acute DENV infection	***Undifferentiated acute febrile illness (AFI)***: studies for which acute dengue infection is not differentiated by clinical grounds alone; IgG prevalence measures obtained during the acute phase of illness is these studies are presumed to reflect secondary infection.
	***Suspected dengue infection***: studies in which defined or undefined clinical criteria for probable dengue infection is stated as an inclusion criterion in the study.

### Risk of bias assessment

In order to gain a better understanding of the quality of prevalence studies identified through the systematic review, the risk of bias (ROB) was assessed for each study based on the Cochrane approach [25] and by evaluating the precision of the reported measures. The methodology for this assessment is similar to that which we have previously developed for reviews of HIV and hepatitis C prevalence in the MENA region [26–28]. Each DENV prevalence measure was considered to have a low, high, or unclear ROB in three domains: sampling methodology, DENV infection ascertainment, and response rate. The latter was defined as the number of tested individuals divided by the number of persons invited to participate in the study [29]. ROB was considered low if (1) sampling was probability-based (i.e. using some form of random selection), (2) DENV prevalence measures included viral neutralization testing (VNT) for general prevalence studies or biological assays (i.e. cell culture, PCR, and NS1 ELISA) for acute infection studies, and (3) response rate was ≥80%. Studies with missing information for any of the domains were classified as having unclear ROB for that specific domain. Sampling strategy was not evaluated for acute infection studies because these studies enrolled individuals presenting to a health facility with acute infection, hence, no population-based sampling is needed to capture this population. Studies were considered to have high precision if the number of individuals tested was ≥ 100. We considered this to be a reasonably sensitive cutoff for precision given the heterogeneous epidemiology of DENV across the region (e.g. a prevalence of 1% entails a 95% CI of 0–3%).

### DENV outbreaks and *Aedes* distribution

To supplement the epidemiologic data gathered through the systematic search, reported outbreaks and *Ae*. *aegypti* or *Ae*. *albopictus* occurrence in the MENA were also sought from the articles retrieved through the search databases as well as through ProMED-MENA and Google Scholar. Given that the characteristics and definitions of dengue outbreaks in the literature are implicitly variable and that there is currently no consensus on how to define such events [30], we broadly included any outbreak report if the author of the report defined the event as an outbreak. Multiple reports of the same outbreak were recorded only once. We manually marked the location of reported outbreaks on the map as well, designating one mark per each first-level administrative division in which one or more outbreaks were identified. In a separate map, we mapped the country-level occurrence of *Ae*. *aegypti* and *Ae*. *albopictus* in order to further inform the existing or potential epidemiology of DENV in the MENA.

## Results

### Search results

**Fig 1:**
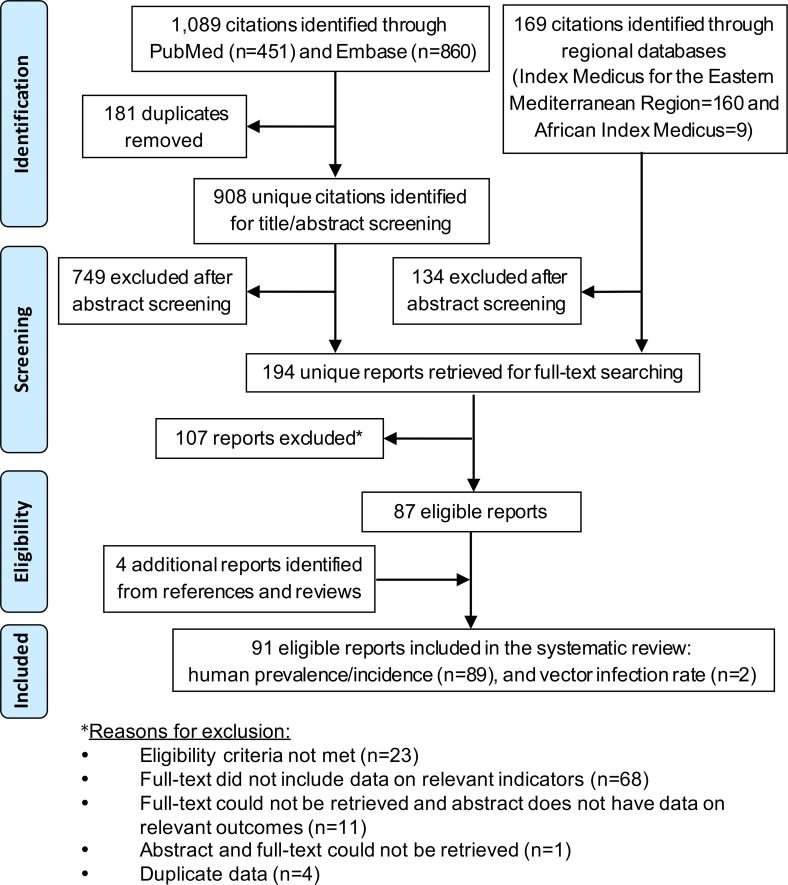
PRISMA flow diagram of article selection. Flow diagram for dengue prevalence, incidence, and vector infection rates in the Middle East and North Africa.

The selection process based on PRISMA guidelines is illustrated in Fig 1 [22]. Briefly, the DENV search yielded 1,258 citations, 91 of which were ultimately eligible for inclusion in the study following the addition of 4 reports identified from the bibliographies of relevant reports and reviews. Four studies from the 1970-80s were excluded that contained DENV seroprevalence of 0–11% in wild and domestic animals in Pakistan, Tunisia, and Turkey, though these may have represented cross-reactions with other flaviviruses [31–34].

### Characteristics of included studies

**Fig 2:**
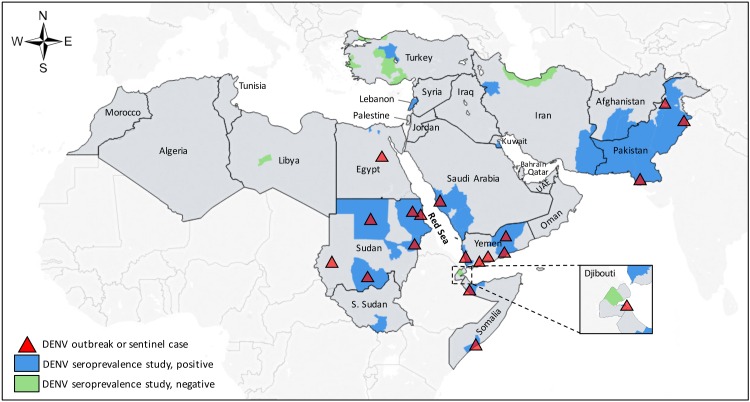
Geographic distribution of human prevalence studies and reported outbreaks of dengue in the Middle East and North Africa.

A total of 105 human prevalence studies for DENV were identified from eligible reports (Table 3). These studies covered 13 of 24 MENA countries and were conducted from 1962–2015. The geographic distribution of these studies is illustrated in Fig 2, and Table 4 contains a frequency summary of these studies. Anti-DENV antibodies were detected in 12 of 13 countries in which studies were reported with a single 1973 study from Libya reporting 0% seroprevalence [35]. The highest number of studies were reported from Pakistan (n = 32) and Sudan (n = 16), most of which targeted populations with acute DENV infection (undifferentiated AFI or suspected dengue infection). Among general population studies, IgG prevalence measures ranged from 0% to 61% and were reported from Djibouti (n = 4, 0–21%), Egypt (n = 4, 0–7%), Iran (n = 3, 0–7%), Kuwait (n = 3, 0–56%), Lebanon (n = 3, 0–61%), Pakistan (n = 3, 9–28%), Saudi Arabia (n = 4, 0–33%), and Sudan (n = 5, 9–49%). ELISAs were the most commonly used diagnostic method for all study types and the majority studies from the MENA used in-house assays (Table 4). VNT results were reported in 3% (n = 3) of all studies while observed or potential serologic cross-reactions with other flaviviruses were present in multiple studies [36–38] (Tables 3 and 4). Three human incidence measures for DENV were identified (Table 5): the first reported an ELISA IgM incidence of 35 cases per 10,000 people living in urban homes in Port Sudan City, Sudan where DENV-carrying mosquitoes were identified over an 11-month period [39]; the second reported an ELISA IgM incidence of 94 cases per 10,000 people in a general population in Port Sudan, Sudan over a 17-week period in 2010 [40]; the third reported an ELISA IgM, NS1 antigen, or PCR incidence of 185 cases per 100,000 febrile children in an urban slum in Karachi, Pakistan from 1999–2001 [41]. Three vector infection rate studies for *Ae*. *aegypti* and *Ae*. *albopictus* were identified from Pakistan and Yemen [42, 43] (Table 6).

**Table 3:** Human prevalence studies for dengue virus in the Middle East and North Africa (n = 105).

Country, Ref.	Year(s) of study*	City or governorate	Setting; population (age range, years)	Sampling	Assay type	Assay make^†^	Target Protein	Assay serotype	Sample size	Prevalence	Additional testing& Comments
**Afghanistan** (n = 1)											
Elyan [44]	2008–10	Uruzgon, Helmand, Kandahar	Hospital; AFI patients (20–59)	Conv.	ELISA	PanBio	Env	1–4	913	19.2%**	2.6% (8/312) were IgM+; observed cross-reaction to WNV, TBEV
**Djibouti** (n = 6)											
Salah [45]	1987	Djibouti City	Military; healthy soldiers	Conv.	IIFA	In-house	wv	2	50	**0%**	
		Randa	Rural community; general pop.	Conv.	IIFA	In-house	wv	2	69	**0%**	
		Djibouti City	Hospital; AFI patients	Conv.	IIFA	In-house	wv	2	41	**0%**	
Rodier [37]	1991	Djibouti City	Clinical setting; AFI patients (1–55)	Conv.	ELISA IgM	In-house	wv	1	91	7.7%**	3.7% (1/27) were VNT+; multiple observed cross-reactions
				Conv.	ELISA IgM	In-house	wv	2	*same*	25.2%**	11.1% (3/27) were VNT+; multiple observed cross-reactions
				Conv.	ELISA IgM	In-house	wv	3	*same*	16.4%**	multiple observed cross-reactions
				Conv.	ELISA IgM	In-house	wv	4	*same*	18.7%**	multiple observed cross-reactions
Fauld [46]	2011	Djibouti City	Animal quarantine station; workers	Conv.	IIFA	EuroImmun	wv	1–4	10	10.0%**	not cross-reactive to WNV
Andayi [47]	2010–11	Djibouti City	Community; general pop. (<1–100)	SRS	ELISA	PanBio	Env	1–4	911	**21.8%**	
**Egypt** (n = 5)											
Mohammed [48]	1966	Abyss	rural community; general pop.	Conv.	HI	In-house	wv	1	29	7.0%**	possible cross-reaction to WNV
					HI	In-house	wv	4	*same*	3.0%**	possible cross-reaction to WNV
		Alexandria	urban community; general pop.	Conv.	HI	In-house	wv	1	55	4.0%**	possible cross-reaction to WNV
					HI	In-house	wv	4	*same*	5.0%**	possible cross-reaction to WNV
Mohammed [49]	1968	Alexandria	Hospital; AFI patients (3–13)	Conv.	HI, CF	In-house	wv	1	120	0%**	0% (0/48) were convalescent +
		Alexandria	Clinical setting; adults	Conv.	HI	In-house	wv	1	78	**0%**	
Darwish [50]	1969	Multiple	University; students	Conv.	HI	In-house	wv	1	1133	**0.3%**	
**Iran** (n = 4)											
Saidi [51]	1970	Multiple	n/s	n/s	HI	In-house	wv	1,2,3	394	6.0%**	possible cross-reaction to WNV
Saidi [52]	1970–71	Caspian region	Community; children (1–6)	Conv.	HI	In-house	wv	2	100	**0%**	
Chinikar [53]	2000–12	Countrywide	Clinical setting; AFI patients	Conv.	ELISA	Vircell	wv	1,2	300	**3.3%**	3.3% (10/300) were IgM+; DEN-1,2 were positive by PCR
Aghaie [14]	2014	Sistan-Baluchestan	blood donor center; general pop.	Conv.	ELISA	PanBio	Env	1–4	540	**7.6%**	78% (32/41) ELISA+ were IFA+
**Kuwait** (n = 8)											
Ibrahim [54]	1966–68	Multiple	Multiple settings; blood donors, non-AFI patients, children (1–60)	Conv.	HI	In-house	wv	1	627	6.5%**	not cross-reactive to DEN-2 or WNV
					HI	In-house	wv	2	*same*	8.1%**	not cross-reactive to DEN-1 or WNV
Al-Nakib [55]	1979–82	Jabriya	Hospital; non-AFI patients (0–60+)	SRS	HI	In-house	wv	1	502	3.2%**	not cross-reactive to DEN-2 or WNV
					HI	In-house	wv	2	*same*	8.4%**	all were cross-reactive to DEN-1, WNV, or TBEV
Pacsa [56]	2002*	Multiple	n/s; Kuwaiti nationals	n/s	ELISA and IgG blot	CDC and Genlab	wv	1–4	425	**13.9%**	only DENV 1–3 were positive
			n/s; Kuwait Bedouins	n/s	ELISA and IgG blot	CDC and Genlab	wv	1–4	47	**0%**	
			n/s; expatriates from South Asia	n/s	ELISA and IgG blot	CDC and Genlab	wv	1–4	266	**37%**	only DENV 1–3 were positive
			n/s; expatriates from Southeast Asia	n/s	ELISA and IgG blot	CDC and Genlab	wv	1–4	31	**56.6%**	only DENV 1–3 were positive
			n/s; expatriates from Middle East	n/s	ELISA and IgG blot	CDC and Genlab	wv	1–4	140	**25%**	only DENV 1–3 were positive
			Hospital; returned travelers with dengue-like illness	n/s	ELISA IgM	PanBio	Env	1–4	210	**9.0%**	only DENV 1–3 were positive; 10%(2/19) IgM+ were PCR+
**Lebanon** (n = 3)											
Garabedian [5]	1962–65	Multiple	Community; general pop. (0–41+)	SRS	HI	In-house	wv	2	113	61.9%**	observed cross-reaction with WNV, YFV
		Multiple	Community; general pop. (0–41+)	SRS	HI	In-house	wv	1	171	49.1%**	observed cross-reaction with WNV, YFV
Hatem [57]	1969	Beirut	n/s	n/s	HI	In-house	wv	2	126	**0%**	
			n/s	n/s	HI	In-house	wv	1	*same*	4.0%**	observed cross-reaction with WNV
**Libya** (n = 1)											
Darwish [35]	1973	Sebha	community and clinic; children, non-AFI patients	Conv.	HI	In-house	wv	1	148	**0%**	
**Pakistan** (n = 32)											
Darwish [31]	1983*	Karachi	Hospital; patients	Conv.	CF	In-house	wv	1	43	**9.3%**	
Akram [58]	1994	Karachi	Hospital; AFI patients (<1–12)	Conv.	ELISA IgM	In-house	wv	1	92	9.8%**	12% (3/25) additional convalescent sera were +; observed cross-reaction to WNV
				Conv.	ELISA IgM	In-house	wv	2	*Same*	14.6%**	24% (6/25) additional convalescent sera were +; observed cross-reaction to WNV
Siddiqui [41]	1999–2001	Karachi	Community; AFI patients (<16)	Conv.	ELISA IgM	Diag. Auto.	wv	1–4	341	**15.8%**	
Tariq [59]	2003	Mangla, Mirpur	Community; suspected dengue	Conv.	ELISA IgM	In-house	n/s	n/s	52	**73%**	
Jamil [60]	2005	Karachi	Hospitals; suspected dengue	Conv.	ELISA IgM	Chemicon	n/s	n/s	106	**36.8%**	
Khan [61]	2006	Karachi	Hospital; suspected dengue (2–72)	Conv.	ELISA IgM	PanBio	Env	1–4	83	**83.6%**	
				Conv	ELISA IgM	Calbiotech	PA	1–4	*same*	**50.7%**	87.8% (73/83) were PCR+ for DEN-2,3 only
Khan [62]	2006	Karachi	Hospital; suspected dengue	Conv.	ELISA	PanBio	Env	1–4	250	**23.2%**	53.6% (134/250) were IgM+; 74% (185/250) were PCR+ for DEN-2 or 3
Koo [63]	2006–11	Multiple	Clinic settings; suspected dengue	Conv.	PCR	In-house		2,3	200	**47%**	none were DEN-1 positive
Khan [64]	2006–07	Hyderabad	Hospital; suspected dengue (13–70)	Conv.	ELISA IgM	In-house	n/s	n/s	50	**40%**	
Khan [65]	2006–07	Multiple	Hospital; suspected dengue	Conv.	ELISA IgM	Calbiotech	PA	1–4	15,040	**26.3%**	
Abbasi [66]	2007–08	Karachi	Hospital; suspected dengue	Conv.	ELISA IgM	Commercial	n/s	n/s	114	**69.6%**	
Tahir [67]	2008	Lahore	Hospital; suspected dengue	Conv.	ICT (IgM)	In-house	n/s	n/s	3215	**54.9%**	
Murad [68]	2008	Shangla	Community; suspected dengue (1–80)	Conv.	ELISA IgM	n/s	n/s	n/s	70	**17.1%**	
Mahmood [69]	2008	Lahore	Hospital; suspected dengue secondary infection (age 1–80)	Conv.	ELISA	NovaLisa	Env	1–4	200	**39.5%**	
			Hospital; suspected dengue primary infection (age 1–80)	Conv.	ELISA IgM	DRG	n/s	2	341	**48.7%**	
Kidwai [70]	2008–09	Karachi	Hospital; suspected dengue (>13)	Conv.	ICT (IgG)	In-house	wv	1–4	599	**83.2%**	41.9% (251/599) were IgM+
Zafar [71]	2009	Rawalpindi	rural communities; adults without history of flavivirus vaccination (>18)	StRS	ELISA	Omega	PA (DEN-2)	1–4	96	**19.8%**	
Zafar [72]	2009	Rawalpindi	Community; general pop.	Conv.	ELISA	Omega,Vircell	PA (DEN-2)	1–4	244	**28.8%**	
Qureshi [73]	2010–12	Karachi	Hospital; suspected dengue	Conv.	ICT (IgM)	In-house	n/s	n/s	162	**9.9%**	
Khan [74]	2010	Punjab	Hospital; suspected dengue (4–60)	Conv.	ELISA IgM	n/s	n/s	n/s	125	**54.4%**	
Hasan [75]	2010	Karachi	Hospital; suspected dengue (>12)	Conv.	ELISA IgM	n/s	n/s	n/s	259	**34.8%**	
Umar [76]	2010	Rawalpindi	Hospital; suspected dengue	Conv.	ELISA IgM	n/s	n/s	n/s	500	**6.8%**	
Jameel [77]	2010	Lahore	Hospital; suspected dengue	Conv.	ELISA IgM	In-house	n/s	n/s	341	**48.7%**	
Naeem [78]	2011	Lahore	Hospital; suspected dengue (1–10+)	Conv.	ELISA IgM	n/s	n/s	n/s	79	**25.3%**	
Ahmed [79]	2011	Lahore	Hospital; suspected dengue (13–81)	Conv.	ELISA IgM	n/s	n/s	n/s	640	**43.9%**	
Ijaz [80]	2011	Lahore	Hospital; suspected dengue (<15–60+)	Conv.	ELISA	n/s	n/s	1–4	5,274	**49%**	
Rashid [81]	2011	Lahore	Hospital; suspected dengue (<18)	Conv.	ELISA	n/s	n/s	n/s	254	**36.6%**	53.9% (137/254) were IgM+
Khan [82]	2011	Lahore	Hospital; suspected dengue (5–50+)	Conv.	ELISA	In-house	wv	1–4	50	**72%**	30% (30/50) were IgM+; 66% (33/50) were PCR+ for DEN-1,2; 60% (30/50) were cell culture+
Hasan [83]	2007–13	Multiple	Hospitals; suspected Crimean-Congo Hemorrhagic Fever	Conv.	ELISA IgM	PanBio	Env	1–4	168	**33.9%**	2.3% (4/168) were PCR+
Ali [84]	2011	Khyber Pakhtunkhwa	Clinical settings; suspected dengue (<10 to >51)	Conv.	ELISA	Diag. Auto.	wv	1–4	612	**20.2%**	31.9% (195/612) were IgM+
Hisam [85]	2012	Rawalpindi	Military Hospital; AFI patients	PS	ELISA IgM	n/s	n/s	n/s	500	**3.2%**	
Assir [86]	2012	Lahore	Hospital; suspected dengue (12–90)	Conv.	ELISA IgM	GmbH	wv	1–4	85	**43.5%**	20% (3/15) were PCR + for DEN-2
**Saudi Arabia** (n = 11)											
Fakeeh [87]	1994–99	Jeddah	Hospitals; suspected dengue (1->50)	Conv.	IIFA, HI	In-house	wv	1,2	985	**31.9%**	16.2% (160/985) were ELISA IgM+; 21% (207/985) were PCR+ (DEN-1,2,3)
Fakeeh [88]	1994–2002	Jeddah	Hospitals; suspected dengue	Conv.	IFA, HI	In-house	wv	1,2,3	1020	**50.5%**	10.8% (110/1020) were ELISA IgM+; 20.5% (209/1020) were PCR+ (DEN-1,2,3)
Khan [89]	2004	Makkah	Hospital; suspected dengue (6–94)		ELISA	PanBio	Env	1–4	136	**32.4%**	58.8% (80/136) were IgM+; 28.1% (27/96) were PCR + (DEN-2,3)
Ayyub [90]	2004–05	Jeddah	Hospital; suspected dengue (2–60)	Conv.	ELISA IgM	n/s	n/s	n/s	80	**48.8%**	
Shahin [91]	2006–08	Makkah	Hospital; suspected dengue	Conv.	ELISA IgM and/or PCR	n/s	n/s	n/s	159	**100%**	
Said [92]	2006	Jeddah	Hospital; suspected dengue (2–71)	Conv.	ELISA IgM	In-house	n/s	n/s	525	**19.2%**	% includes paired serum sample
Memish [93]	2010	Multiple	Military; adults	Conv.	ELISA	PanBio	Env	1–4	1024	**0.1%**	0% of IgG+ were IgM+
Gamil [94]	2010–11	Jeddah	Hospitals; suspected dengue (3–56)	Conv.	n/s	n/s	n/s	n/s	553	**47.7%**	
Al-Azraqi [95]	2013	Jizan	Clinics; clinic attendants (1–60+)	SRS	ELISA	Focus	wv	1–4	268	**26.5%**	
		Aseer	Clinics; clinic attendants (1–60+)	SRS	ELISA	Focus	wv	1–4	697	**33.7%**	
Ashshi [96]	2014	Mecca	blood donation center; adults	Conv.	ELISA	PanBio	Env	1–4	100	**7%**	6% (6/100) were IgM+;1% (1/100) were NS1+
**Somalia** (n = 7)											
Botros [97]	1987	Hargeysa	Refugee camp; AFI patients	Conv.	ELISA	In-house	wv	2	38	**60.7%**	acute and convalescent samples; 39.4% (15/38) were IFA+; 37.9% (11/29) were HI+; 14.2% (4/28) were ELISA IgM+
Kanesa-thasan [98]	1993	n/s	Military base; AFI soldiers	Conv.	ELISA IgM and/or HI	n/s	n/s	n/s	84	**17.8%**	93% (14/15) were cell culture + (DEN-2 and 3 only)
Sharp [99]	1992–93	Mogadishu	Military Hospital; AFI patients (soldiers)	Conv.	ELISA IgM	In-house	wv	1–4	129	**34.9%**	40.6% (39/96) were cell culture positive for DEN-2; 2% (2/96) were cell culture positive for DEN-3
		Baardera	Military; adults (19–25)	Conv.	ELISA IgM	In-house	wv	1–4	494	7.7%**	observed cross-reaction with WNV
Nur [100]	1995	Mogadishu	Hospital; children (<1 to > 2 years of age)	CC.	ELISA IgM	Progen	wv	2	23	**0%**	
			Hospital; AFI patients with / without rash (<1 to > 2 years of age)	CC.	ELISA IgM	Progen	wv	2	46	**0%**	
Kyobe Bosa [101]	2011	Mogadishu	Hospitals; AFI patients (20–49)	Conv.	ELISA IgM	n/s	n/s	1,2,3	134	**80%**	62% (83/134) were PCR+
**Sudan** (n = 16)											
Omer [36]	1976	Gezira	Rural community; general pop. (5–40+)	Conv.	HI	In-house	wv	2	109	**27.5%**	17.4% (19/109) were VNT+
Hyams [102]	1984	Port Sudan	Hospital; AFI patients (12–70)	Conv.	HI	In-house	wv	n/s	100	**3%**	14.8% (8/54) were convalescent +; 1% (1/100) DEN-1 cell culture +; 17% (17/100) DEN-2 cell culture +
Woodruff [103]	1986	Juba	Hospital; patients with history of fever within past 6 months and AFI patients (1–85)	Conv.	HI	In-house	n/s	n/s	130	40.0%**	represents single virus activity not cross-reactive to multiple flaviviruses tested
McCarthy [104]	1988	Khartoum	Clinical setting; non-AFI patients	CC	ELISA	In-house	wv	2	100	**49%**	0% were IgM+
			Clinical setting; AFI patients (1–89)	CC	ELISA	In-house	wv	2	196	48%**	0% were IgM+; possible cross-reaction to WNV
Watts [18]	1989	Northern Province	Clinical setting; AFI patients (11–70)	Conv.	ELISA	In-house	n/s	2	185	24.0%**	possible cross-reactions to multiple flaviviruses
Ibrahim [105]	1997–99	Khartoum	Clinical setting: suspected measles	Conv.	ELISA IgM	MRL Diag.	n/s	n/s	188	**3.2%**	
Malik [106]	2004–05	Port Sudan	Hospitals; suspected dengue (<1–15)	Conv.	ELISA IgM	PanBio	Env	1–4	40	**90.0%**	39% (9/23) were PCR+ (DEN-3)
Gould [107]	2005	South Kordofan	Clinical setting; suspected YF patients (n = 3), severe illness (n = 8), AFI patients (n = 7), healthy (n = 16)	Conv.	ELISA IgM	In-house	wv	n/s	34	5.9%**	observed cross-reaction with YFV, WNV
Farnon [38]	2005	Kortalla	Community; general pop., YF vaccinated (0–44+)	SSCS	ELISA	In-house	wv	1–4	87	1.1%**	observed cross-reaction in YF vaccine recipient; 0% were IgM+; 52% (45/87) were VNT+ for DENV and YFV
Seidahmed [39]	2008–09	Port Sudan City	Urban community; individuals from houses with DENV-carrying mosquitoes (<1–80)	RSS	ELISA IgM	PanBio	Env	1–4	791	**5.2%**	
Adam [108]	2008–09	Port Sudan City	Hospitals; pregnant women with deliveries	Ret. cohort	ELISA IgM	n/s	n/s	1–4	10,820	**0.7%**	
Himatt [109]	2011	Kassala state	Community; general pop. (5–75+)	MSCS	ELISA	PanBio	Env	1–4	489	**9.4%**	0.6% (3/489) were IgM+
Abdalla [110]	2012	Kassala State	Hospital; AFI patients with suspected measles (2–65)	Conv.	ELISA	PanBio	Env	1–4	60	**11.7%**	
Elduma [15]	2012	Port Sudan	Hospital; pregnant women with AFI	Conv.	ELISA	Commercial	n/s	n/s	39	**12.8%**	2.6% (1/39) were IgM+ and PCR+
Soghaier [111]	2014	South Kordofan	Urban and rural communities; general pop. (15–60)	MSCS	ELISA	PanBio	Env	1–4	600	**27.7%**	77% of study population were YFV vaccinated
**Turkey** (n = 6)											
Ari [34]	1971	Izmir	Community and clinic; general pop.	Conv.	HI	In-house	wv	2	270	**0%**	
Radda [112]	1973*	Izmir	n/s; general pop.	Conv.	HI	In-house	wv	2	270	0.3%**	observed cross-reaction with WNV
		Istanbul	n/s; general pop.	Conv.	HI	In-house	wv	2	90	**0%**	
		Ankara	n/s; general pop.	Conv.	HI	In-house	wv	2	95	**0%**	
Ergunay [113]	2010	Ankara, Konya, Eskisehir, Zonguldak	blood donation center; blood donors	Conv.	ELISA	EuroImmun	wv	1–4	2435	**0.9%**	14.2% (3/21) of IgG+ were IIFT+ for DEN-2; 9.5% (2/21) of IgG+ were IgM+
Tezcan [114]	2010–11	Mersin	blood donation center; blood donors	Conv.	ELISA	Vircell	wv	1–4	920	**16.6%**	0.9% (8/920) were IgM+; 0% were NS1+
**Yemen** (n = 5)											
Bin Ghouth [115]	2011	Hadramout	Hospital; suspected dengue (<5 to 55+)	Conv.	ELISA	PanBio	Env	1–4	982	**50.6%**	64.1% (630/982) IgM+; 86.2% (163/189) PCR+ for DEN-3
Malik [116]	2010–11	Al-Hudaydah	Clinical setting; AFI patients (0–45+)	Conv.	ELISA	PanBio	Env	1–4	136	**87.5%**	8.1% (11/136) were IgM+
Madani [117]	2010	Hadramout	Clinical settings; suspected viral hemorrhagic fever (3–75)	Conv.	ELISA	PanBio	Env	1–4	207	**48.3%**	78.7% (163/207) IgM+; 46.9% (97/207) NS1+; 0.09% (2/207) PCR+ for DEN-1,2
Rezza [118]	2012	Al Hudaydah	Hospitals; AFI patients with dengue-like illness (1–60)	CS	ELISA	NovaLisa	Env	1–4	400	**72.5%**	18% (72/400) IgM+; 13.8% (55/400) PCR+ for DEN-1,2
Qassem [119]	2013	Hadramout	Clinical setting; suspected dengue and/or west nile infection	Conv.	ELISA IgM	n/s	n/s	n/s	42	19.0%**	observed cross-reaction with WNV

* Indicates year of publication when year(s) of data collection not available in report.

^**†**^ All serologic assays were IgG unless otherwise stated.

**Indicates documented occurrence or suspicion of false-positives due to cross-reactions with other same family viruses or low serologic titers.

Abbreviations: AFI, acute febrile illness patients; Ag, antigen; CF, complement fixation; Conv, convenience; ELISA, enzyme-linked immunosorbent assay; HI, hemagglutinin inhibition; ICT, immunochromatography test; IIFA, indirect immunofluorescence antibody test; MSCS, multi-stage cluster sampling; n/s, not specified; NS1, NS1 antigen test; PA, purified antigen; PCR, polymerase chain reaction; pop., population; PS, purposive sampling; RSS, random stratified sampling; SRS, simple random sampling; SSCS, single stage cluster sampling; VNT, viral neutralization test

Assay Abbreviation: *CDC* (Centers for Disease Control and Prevention, USA); *Chemicon* (Chemicon, Temecula, CA, USA); *Diag*. *Auto*. (Diagnostic Automation, CA, USA); *DRG* (DRG International Inc); *Euroimmun* (Lubeck, Germany); *Focus* (Focus Diagnostics, Cypress CA, USA); Genlab (Genlab Diagnostics, Singapore); GmbH (Human GmbH, Wiesbaden, Germany); *MRL Diagnostics* (Cypress CA, USA); *NovaLisa* (Dietzenbach, Germany); *Omega* (Omega Diagnostics, Scotland, UK); *PanBio* (Brisbane, Australia); *Progen* (Heidelberg, Germany); *SD Bioline* (Standard Diagnostics, Korea); *Vircell* (Vircell Microbiologists, Granada, Spain)

**Table 4:** Summary of human prevalence studies for dengue virus in the Middle East and North Africa (n = 103).*

**Study characteristics**	**General population (n = 42) n (%)**	**Acute febrile illness (n = 23) n (%)**	**Suspected dengue (n = 38) n (%)**
Total sample size	24,377	4,065	33,955
Median DENV % prevalence (range %)	25% (0–61.9)	15.2% (0–87.5)	47.4% (6.8–100)
Year of study			
before 1990	21 (50%)	7 (30%)	0
1990 to 2015	21 (50%)	16 (70%)	38 (100%)
Study setting			
community	31 (74%)^**†**^	2 (9%)	2 (5%)
clinic or hospital	11 (26%)	21 (91%)	36 (95%)
Assay			
ELISA IgG	19 (45%)	8 (35%)	10 (26%)
ELISA IgM	11 (26%)	17 (74%)	31 (82%)
immunofluorescence antibody	5 (12%)	2 (9%)	2 (5%)
hemagglutination inhibition	15 (36%)	5 (22%)	2 (5%)
complement fixation	1 (2%)	1 (4%)	0
viral neutralization	2 (5%)	1 (4%)	0
PCR	0	4 (17%)	14 (37%)
cell culture	0	3 (13%)	1 (3%)
NS1 antigen	2 (5%)	0	1 (3%)
Assay make			
in-house	21 (50%)	10 (43%)	11 (29%)
commercial	20 (48%)	10 (43%)	15 (39%)
not specified	1 (2%)	3 (13%)	12 (32%)
Target protein**			
whole virus	32 (76%)	12 (52%)	6 (16%)
envelope	7 (17%)	4 (17%)	9 (24%)
not specified	3 (7%)	7 (30%)	21 (55%)
**Risk of bias summary**			
Assay			
low risk of bias	2 (5%)	8 (35%)	14 (37%)
high risk of bias	40 (95%)	15 (65%)	24 (63%)
unclear risk of bias	0	0	1 (3%)
Sampling methodology			
low risk of bias	15 (36%)	n/a	n/a
high risk of bias	17 (40%)	n/a	n/a
unclear risk of bias	10 (24%)	n/a	n/a
Response rate			
low risk of bias	6 (14%)	11 (48%)	22 (58%)
high risk of bias	1 (3%)	0	0
unclear risk of bias	35 (83%)	12 (52%)	16 (42%)
Precision			
High	28 (67%)	15 (65%)	28 (74%)
Low	14 (33%)	8 (35%)	10 (26%)

* N = 103 because the study type (i.e. general prevalence, acute febrile illness, or suspected dengue) was not specified in two studies [51, 57].

^**†**^Community study settings also include animal quarantine station (n = 1), blood donation center (n = 5), military (n = 3), and university (n = 1).

** Indicates the target protein for the initial screening assay for studies in which multiple diagnostic assays were utilized.

**Table 5:** Summary of human incidence studies for dengue virus in the Middle East and North Africa (n = 3).

Country, Ref.	Year(s) of study	Duration of follow-up	City or governorate	Setting; population(age range, years)	Study design	Sampling	Assay type	Assay make^+^	Assay Target	Serotype tested	Sample size	Incidence
**Pakistan** (n = 1)												
Siddiqui [41]	1999–2001	1999–2001	Karachi	Urban slum; children <16 years of age with undifferentiated febrile illness	CS	Active surveillance	ELISA IgM	Diag. Auto.	wv	1–4	1,248	**185/100,000**
**Sudan** (n = 2)												
Seidahmed [39]	2008–09	12 months	Port Sudan City	Urban community; general pop. living in houses where DENV-carrying mosquitoes were present (<1–80)	Pros. coh	RSS	ELISA IgM	PanBio	Env	1–4	791	**35/10,000**
Seidahmed [40]	2010	17 weeks	Port Sudan	Urban community; general pop.	CS	Conv.	ELISA IgM, NS1, PCR	n/s	n/s	n/s	3,765^‡^	**94/10,000**

^‡^ Reported cases

Abbreviations: CS, cross-sectional; ELISA, enzyme-linked immunosorbent assay; Env, envelope; PCR, polymerase chain reaction; Pros. coh, prospective cohort; RSS, random stratified sampling

Assay Abbreviation: *Diag*. *Auto*. (Diagnostic Automation, CA, USA); *PanBio* (Brisbane, Australia)

**Table 6:** Summary of vector infection rate studies for dengue virus in the Middle East and North Africa (n = 3)

Author, Ref.	Year(s) of data collection	City or governorate	Setting	Mosquito species	Assay type	Sample size	Infection rate	Comments
**Pakistan**									
Jahan [42]	2011	Lahore	Urban areas	*Ae*. *aegypti*	Ag-capture ELISA	114 pools (n = 570 mosquitoes)	**27.2%**	
				*Ae*. *albopictus*	Ag-capture ELISA	4 pools (n = 20 mosquitoes)	**25%**	
**Yemen**								
Zayed [43]	2010–11	Al Hodayda	houses of CHIKV cases at Eritrean refugee camp	*Ae*. *aegypti*	RT-PCR	11 pools (n = 30 mosquitoes)	**0%**	17 *Culex spp*. mosquitoes were also negative for DENV RNA.

Abbreviations: RT-PCR, reverse transcription-polymerase chain reaction

### Risk of bias assessment results

The quality assessment for each study is found in S1 Table and a summary of the precision and risk of bias assessment is found in Table 4. In brief, most studies (≥65%) contained high precision as defined by a sample size of ≥100 participants. A minority (36%) of general population seroprevalence studies utilized some form of random sampling, and response rates were either <80% or not reported in 86% of general population studies. VNT or a biologic confirmatory assay (i.e. cell culture, PCR, and NS1 ELISA) was performed in 5% and 36% of general population seroprevalence and acute DENV infection studies, respectively, entailing low ROB for the assays used.

### Dengue outbreaks and *Aedes* occurrence

Reported outbreaks of DENV in the region were gathered through citations collected from the search databases (S2 Table) and mapped along with the geographic distribution of prevalence studies in Fig 2. For DENV, 81 outbreaks were reported from 9 countries in the region from 1941–2015, including sentinel reports of autochthonous transmission in Egypt (2010) and Yemen (1983). Reports contained variable descriptions of outbreaks including ‘estimated’, ‘suspected’, ‘reported’, and/or laboratory ‘confirmed’ cases (S2 Table). The definition that qualified each event as an outbreak was unclear in most instances. Outbreaks of DENV serotypes 1–3 were reported from countries surrounding the Red Sea and DENV-4 was only reported from Pakistan [120, 121]. Although, in general, DENV serotypes were not reported consistently.

**Fig 3:**
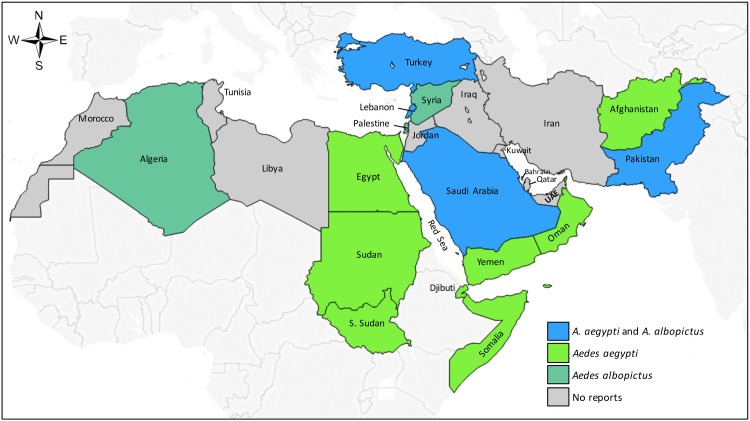
Country-level distribution of *Aedes aegypti* and *Aedes albopictus* occurrence in the Middle East and North Africa.

Published reports of *Ae*. *aegypti and Ae*. *albopictus* occurrence are recorded in S3 Table and mapped by country in Fig 3. *Ae*. *aegypti* occurrence was reported in 11 MENA countries and historically (i.e. prior to 1960) in Algeria, Libya, Morocco, Syria, and Tunisia. *Ae*. *albopictus* was reported in seven MENA countries, including Algeria, Palestine, and Syria, countries where *Ae*. *aegypti* is not currently reported. No published reports of *Ae*. *aegypti* or *Ae*. *albopictus* occurrence (or DENV outbreaks) were identified in seven MENA countries: Bahrain, Iran, Iraq, Jordan, Kuwait, Qatar, and United Arab Emirates. Since 2005, *Ae*. *aegypti* and/or *Ae*. *albopictus* occurrence has been documented in Afghanistan, Algeria, Lebanon, Oman, Palestine, Syria, and Turkey, though autochthonous transmission of DENV has not yet been reported from these countries.

## Discussion

Our study offers an assessment of published prevalence, incidence, and outbreak reports pertaining to the epidemiology of dengue in the MENA region. Based on the study results, the MENA contains two apparent subregions known to harbor DENV: 1) Pakistan, and 2) the Red Sea countries (Djibouti, Egypt, Saudi Arabia, Somalia, Sudan, and Yemen). No seroprevalence or outbreak data was identified across broad areas of the MENA, however, including some *Aedes* endemic areas. There was also a paucity of reports estimating human incidence and vector infection rates. These findings suggest priorities for future research. However, they also challenge efforts to synthesize and compare the inter- and intra-country epidemiology of DENV in the region.

### Dengue seroprevalence in the MENA

In our review, Pakistan reported the highest number of prevalence studies and the broadest study coverage among MENA countries. Multiple studies reported >20% prevalence in both general population and those with undifferentiated AFI [1, 106, 122–124]. DENV serotypes 1–4 are known to circulate in Pakistan, unlike other MENA countries [82]. Pakistan also reported the largest number of confirmed cases among all DENV outbreaks in the MENA, with 21,580 cases reported during the 2011 DENV-2 outbreak [79, 120] (S2 Table).

In the Red Sea region, multiple general population and AFI population IgG seroprevalence measures exceeding 20% were published from in Djibouti, Saudi Arabia, Somalia, Sudan, and Yemen within the past decade (Table 3) along with multiple confirmed outbreaks of DENV serotypes 1–3 since the 1980s (S2 Table). DENV-4 has not yet been identified in this subregion to our knowledge. Although reported outbreaks and cases often localize along the Red Sea coastline in these countries [1], seroprevalence studies suggest a broader distribution of DENV infections that are likely underdetected (Fig 2). This is illustrated by the sentinel report of a DENV-infected traveler returning from Yemen in 1983 [125], despite the first outbreaks of DENV in Yemen and Saudi Arabia not being reported until 1994 [126–128]. Our search also identified no published prevalence studies or outbreaks in Egypt after 1969 until a dengue outbreak was reported in November 2015 [10]. However, DENV transmission was suggested years prior by a report of two travelers diagnosed with dengue after returning from southern Egypt in 2011 [129] and the identification of *Ae*. *aegypti* in southern Egypt that same year [130]. It is plausible that undetected DENV transmission had been occurring in Egypt prior to this outbreak. However, it is not clear whether this and other recent outbreaks represent increasing incidence, increasing detection, or both, amidst the heterogeneity in study coverage and reporting in the MENA.

### Clinical and methodological diversity among studies

An important finding in our study was the clinical and methodological diversity among DENV prevalence studies. This diversity represents a challenge to synthesizing the epidemiologic literature for DENV in the MENA. Clinically, studies represented a diversity of human populations of different ages and demographics, in different years, and different locations and transmission contexts. Ninety-six percent of studies from Afghanistan, Pakistan, Saudi Arabia, Somalia, and Yemen were conducted during or prior to 1990. However, 53% of studies in other MENA countries were conducted prior to 1990, when study methods and DENV epidemiology may have been different. Methodologically, most studies utilized convenience samples without reporting response rates, entailing high risk of bias and uncertainty in the representativeness of reported measures (Table 4). These findings, along with the high variability in regional study coverage, precluded meta-analyses of the available data.

### Flavivirus cross-reactivity

Serologic cross-reactions remain a challenge to seroepidemiologic studies for DENV and other flaviviruses. Viral neutralization tests, considered the gold standard serologic assay for DENV, were performed in only 5% of general population seroprevalence studies in our review. Compared to ELISAs, seroprevalence measures were 22–86% lower by secondary/confirmatory testing with immunofluorescence or VNT in our review [14, 36, 37, 113]. This illustrates the potential uncertainty surrounding the reliability of ELISAs in DENV serologic studies, particularly in areas where the prevalence of antigenically similar viruses is broad or unknown. West Nile virus (WNV), for example, is thought to be distributed across the MENA on account of its ubiquitous *Culex spp*. vector and migratory bird flyways [131–133]. With up to 80% of WNV infections occurring subclinically, the potential for serologic cross-reactions with DENV antibody assays must be considered. Yellow fever vaccine-derived and natural antibodies may also cross-react with anti-DENV antibodies, especially relevant in YFV endemic regions such as Sudan [38, 107, 111]. As the emergence of zika virus in the Western Hemisphere or the re-emergence of YFV has shown, serologic assays with low specificity are inadequate to tackle the epidemiologic challenges of emerging arboviral diseases [134].

### Heterogeneity in dengue outbreak reports

Our review identified DENV outbreaks in over a third of MENA countries, with most outbreaks reported from Pakistan, Sudan, and Saudi Arabia (S2 Table). Outbreaks varied widely across time and space in the MENA: reported cases varied from <10 to >100,000 over a span of months to years, reported from the village level to the level of the province and region. This presents a challenge to epidemiologic monitoring and policy planning for DENV, as use of different outbreak definitions results in differences in early detection and response [30]. There is currently no consensus on how to define DENV outbreaks, and adopting a common definition for the MENA is challenging given the region’s heterogeneous infection pressures, multiple DENV serotypes, and variable surveillance and detection capacity. At present, assessing whether a reported transmission event in the MENA significantly deviates from baseline transmission, and thus constitutes an outbreak, is often unclear.

### Risk factors and research priorities

Our study did not identify confirmed DENV transmission in any of the MENA countries west of Egypt and east of Saudi Arabia until Pakistan (Fig 2). However, the paucity of published data in these sub-regions does not preclude the possibility of unrecognized transmission in some areas or the risk of emergence in others. Indeed, modeling studies suggest ecologic niches for *Aedes* along the coastal Mediterranean Basin of North Africa [1, 123, 135], and *Ae*. *albopictus* and/or *Ae*. *aegypti* has been recently reported in Algeria, Lebanon, Palestine, Syria, and Turkey [5, 136–140] (Fig 3 and S3 Table). In contrast, *Ae*. *albopictus* has been identified along the Mediterranean coast of Europe for decades along with local transmission of DENV and chikungunya since 2007 [141]. Near the Pakistan border, serologic evidence suggests possible DENV transmission in Iran [14, 51, 53] and Afghanistan [44], though local transmission has not been confirmed to our knowledge [53]. The presence of *Aedes* or DENV transmission in these areas should not be ruled out [53].

Several ecologic and social factors in the MENA may promote the spread of *Aedes-*borne viruses like DENV. Urbanization [142] may increase the risk of outbreaks and use of open water storage containers that promote *Ae*. *aegypti* breeding [1, 39, 43, 55, 95, 111, 123, 135]. Heavy rainfall has been implicated in DENV outbreaks in Sudan, Djibouti, and Yemen [12, 47, 143, 144], which may become increasingly unpredictable through climate change [145]. Armed conflicts and economic turmoil in Iraq, Syria, and Yemen may render these areas vulnerable to vector-borne diseases while diminishing surveillance and response [146]. Inter-regional migration poses risk for imported DENV, as millions of migrants travel from DENV-endemic countries to the Arabian Peninsula [111, 126, 146–149] and to Mecca, Saudi Arabia to attend Umra and Hajj [126]. Intra-regionally, heavy commerce in the Red Sea region likely drives DENV serotype mixing and spread [116, 148], as evidenced by multiple DENV outbreaks occurring at port cities in Djibouti [37, 45], Saudi Arabia [126], Sudan [39], and Yemen [116, 148]. Contiguous spread of DENV from Yemen to Oman [150], or from Pakistan to Iran or Afghanistan [14], may also pose risk.

A number of research priorities emerge concerning the epidemiology of DENV in the MENA. First, broader seroepidemiologic coverage in the region is needed. Such studies are efficient means of characterizing infection pressures in populations lacking surveillance and diagnostic capacity. Multiplexed diagnostics are increasingly available and are well-suited for concurrently exploring the distribution other undercharacterized arboviruses in the region (e.g. Alkhumra, Chikungunya, Crimean-Congo Hemorrhagic Fever, O’Nyong-nyong, Rift Valley Fever, Sandfly Fever virus complex, Usutu, and West Nile viruses). Second, serologic studies should include methods to minimize cross-reactions, particularly for flaviviruses [151]. Third, seroepidemiologic studies should incorporate uniformity in study design and enrollment criteria to minimize confounding, such as standard case definitions for studies of ‘suspected’ dengue [126]. Ideally this could include population-based sampling that provides baseline data to benchmark the regional impact of these pathogens over the coming years. Fourth, studies should incorporate vector surveillance and infection rates. Such studies are important for understanding transmission dynamics that inform vector control strategies and predict future transmission and disease risk [123, 135, 141]. Guidelines and tools for calculating vector infection rates are available [141, 152]. Finally, attaining a meaningful definition of DENV outbreaks in the MENA countries will require a thorough assessment of baseline surveillance, control, and treatment capacities in endemic regions [30].

### Study limitations

Our study was limited by its reliance on select databases of peer-reviewed literature screened by one investigator with the exclusion of grey literature which may have provided additional data. Reviewing other *Aedes*-transmitted pathogens or studies reporting *Aedes* distribution in the MENA may also have provided further insights regarding the potential geographic distribution of DENV. Due to the limitations in the content and distribution of studies, we did not perform a meta-analysis nor did we explore bias in overall outcome measures through a funnel plot or Egger test. Non-publication of studies with small or zero effect size or studies targeted to known dengue-endemic areas may have biased the distribution and quantity of DENV studies. The prevalence measures themselves may have been biased through serologic-cross reactions, targeting of older study populations (with higher seroprevalence), and lack of convalescent titers for acute DENV infection studies (possibly underestimating seroprevalence).

## Conclusions

DENV seroprevalence in the MENA is high among some populations in the Red Sea region and Pakistan, while recent outbreaks in these subregions suggest increasing DENV incidence driven by ecologic and social factors. Published prevalence and incidence, vector occurrence, and vector infection rates are lacking in broad areas of the MENA and available studies contain methodological limitations. These findings illustrate the need to strengthen programs for surveillance, reporting, and control of DENV and *Aedes* in the MENA, both to define DENV and *Aedes* epidemiology and to mitigate the risk of emerging *Aedes*-transmitted pathogens in the future.

## Supporting Information

S1 Fig Preferred Reporting Items for Systematic Reviews and Meta-analyses (PRISMA) checklist. (PDF)

S2 Fig Data sources and search criteria used for the systematic review of dengue virus prevalence and incidence in the Middle East and North Africa. (PDF)

S1 Table Precision and risk of bias assessment for dengue prevalence measures in the Middle East and North Africa. (PDF)

S2 Table Summary of reported outbreaks and sentinel cases for dengue virus in the Middle East and North Africa. (PDF)

S3 Table Reported *Aedes aegypti* and *Aedes albopictus* occurrence in the Middle East and North Africa. (PDF)
