# Supplementary material for: Dengue in the Middle East and North Africa: A Systematic Review
Source: PLoS Negl Trop Dis. 2016 Dec 7;10(12):e0005194. doi: 10.1371/journal.pntd.0005194 (PMC5142774; doi:10.1371/journal.pntd.0005194)
Supplement: S2 Fig — (PDF) [file pntd.0005194.s002.pdf]

**Dengue Search Criteria**
**PubMed** (Last searched December 9, 2015)
("Dengue"[text] OR "Dengue"[mesh] OR "Dengue Virus"[mesh] OR “Aden fever”[text] or “bouquet fever”[text] or “breakbone fever”[text] or “dandy fever”[text] or “red fever”[text] or “solar fever”[text] or “sun fever”[text] OR Flavivir*[text] OR “Arbovirus, Group B”[Mesh]) **AND** ("Middle East"[Mesh] OR "Islam"[Mesh] OR "Arabs"[Mesh] OR "Arab World"[Mesh] OR "Africa, Northern"[Mesh] OR "Sudan"[Mesh] OR "Somalia"[Mesh] OR "Djibouti"[Mesh] OR "Pakistan"[Mesh] OR "Middle East"[Text] OR "Middle-East"[Text] OR "North Africa"[Text] OR "North-Africa"[Text] OR "EMRO"[Text] OR "Eastern Mediterranean"[Text] OR “Arab”[Text] OR “Arabs”[Text] OR “Arab World”[Text] OR "Islam"[Text] OR "Afghanistan"[Text] OR "Algeria"[Text] OR "Bahrain"[Text] OR "Djibouti"[Text] OR "Egypt"[Text] OR "Jordan"[Text] OR "Kuwait"[Text] OR "Lebanon"[Text] OR "Libya"[Text] OR "Iran"[Text] OR "Iraq"[Text] OR "Morocco"[Text] OR "Oman"[Text] OR "Pakistan"[Text] OR "Qatar"[Text] OR "Saudi Arabia"[Text] OR "Somalia"[Text] OR "Sudan"[Text] OR "Syria"[Text] OR "Tunisia"[Text] OR “Turkey”[text] OR "United Arab Emirates"[Text] OR "Dubai"[Text] OR "Abu Dhabi"[Text] OR "Abu-Dhabi"[Text] OR “Sharjah”[Text] OR "West Bank"[Text] OR "Ghaza"[Text] OR "Palestine"[Text] OR"Yemen"[Text])
**Embase** (Embase 1974 to 2015 Week 49; Last searched December 9, 2015)
(exp Dengue/ OR Dengue.mp OR Aden Fever.mp OR Bouquet Fever.mp OR Breakbone Fever.mp OR Dandy Fever.mp OR Red Fever.mp OR Solar Fever.mp OR Sun Fever.mp OR Flavivir*.mp OR Arbovirus, Group B.mp.) **AND** (exp Middle East/ or exp North Africa/ or exp Arab/ or exp Afghanistan/ or exp Djibouti/ or exp Pakistan/ or exp Somalia/ or exp Sudan/ or Middle East.mp. or North Africa.mp. or EMRO.mp. or Eastern Mediterranean.mp. or Arab.mp. or Arabs.mp. or Arab World.mp. or Islam.mp. or Afghanistan.mp. or Algeria.mp. or Bahrain.mp. or Djibouti.mp. or Egypt.mp. or Jordan.mp. or Kuwait.mp. or Lebanon.mp. or Libya.mp. or Iran.mp. or Iraq.mp. or Morocco.mp. or Oman.mp. or Pakistan.mp. or Qatar.mp. or Saudi Arabia.mp. or Somalia.mp. or Sudan.mp. or Syria.mp. or Tunisia.mp. or Turkey.mp or United Arab Emirates.mp. or Dubai.mp. or Abu Dhabi.mp. or Sharjah.mp. or West Bank.mp. or Ghaza.mp. or Palestine.mp. or Yemen.mp.)
**Regional Databases**
**Index Medicus for the Eastern Mediterranean Region and African Index Medicus** (Last searched December 9, 2015)
a search was conducted using only the term ‘dengue’
