# Supplementary material for: Dengue in the Middle East and North Africa: A Systematic Review
Source: PLoS Negl Trop Dis. 2016 Dec 7;10(12):e0005194. doi: 10.1371/journal.pntd.0005194 (PMC5142774; doi:10.1371/journal.pntd.0005194)
Supplement: S1 Table — (PDF) [file pntd.0005194.s003.pdf]

S1 Table. Precision and risk of bias assessment for dengue prevalence measures in the Middle East and North Africa.

Country, Ref.	Year(s) of study	Population	Sample size	Prevalence	Risk of Bias Assessment			Precision
					Assay	Sampling*	Response rate	
**Afghanistan**								
Elyan [1]	2008-10	AFI patients	913	**19.2%**	High ROB	n/a	Unclear ROB	High
**Djibouti**								
Salah [2]	1987	healthy soldiers	50	**0%**	High ROB	High ROB	Unclear ROB	Low
		general population	69	**0%**	High ROB	High ROB	Unclear ROB	Low
		AFI patients	41	**0%**	High ROB	n/a	Unclear ROB	Low
Rodier [3]	1991	AFI patients	91	**7.7-25.0%**	Low ROB	n/a	Unclear ROB	Low
Fauld [4]	2011	animal workers	10	**10.0%**	High ROB	High ROB	Unclear ROB	Low
Andayi [5]	2010-11	general population	911	**21.8%**	High ROB	Low ROB	High ROB	High
**Egypt**								
Mohammed [6]	1966	general population	29	**3.0-7.0%**	High ROB	High ROB	Unclear ROB	Low
		general population	55	**4.0-5.0%**	High ROB	High ROB	Unclear ROB	Low
Mohammed [7]	1968	AFI patients	120	**0%**	High ROB	n/a	Unclear ROB	High
		clinic population	78	**0%**	High ROB	Unclear ROB	Unclear ROB	Low
Darwish [8]	1969	university students	1133	**0.3%**	High ROB	High ROB	Unclear ROB	High
**Iran**								
Saidi [9]	1970	n/s	394	**6.0%**	High ROB	Unclear ROB	Unclear ROB	High
Saidi [10]	1970-71	children	100	**0%**	High ROB	Low ROB	Unclear ROB	High
Chinikar [11]	2000-12	AFI patients	300	**1.0-3.3%**	High ROB	n/a	Unclear ROB	High
Aghale [12]	2014	blood donors	540	**7.6%**	High ROB	High ROB	Unclear ROB	High
**Kuwait**								
Ibrahim [13]	1966-68	blood donors, patients, children	627	**6.5-8.1%**	High ROB	Low ROB	Unclear ROB	High
Al-Nakib [14]	1979-82	patients	502	**3.2-8.4%****	High ROB	Low ROB	Unclear ROB	High
Pacsa [15]	2002*	general population	425	**13.9%**	High ROB	Unclear ROB	Unclear ROB	High
		general population	47	**0%**	High ROB	Unclear ROB	Unclear ROB	Low
		expatriates from South Asia	266	**37%**	High ROB	Unclear ROB	Unclear ROB	High
		expatriates from Southeast Asia	31	**56.6%**	High ROB	Unclear ROB	Unclear ROB	Low
		expatriates from Middle East	140	**25%**	High ROB	Unclear ROB	Unclear ROB	High
		suspected dengue	210	**9.0%**	Low ROB	n/a	Unclear ROB	High
**Lebanon**								
Garabedian [16]	1962-65	general population	113	**61.9%**	High ROB	Low ROB	Unclear ROB	High
		general population	171	**49.1%**	High ROB	Low ROB	Unclear ROB	High
Hatem [17]	1969	n/s	126	**0%**	High ROB	Unclear ROB	Unclear ROB	High
		n/s	126	**4.0%**	High ROB	Unclear ROB	Unclear ROB	High
**Libya**								
Darwish [18]	1973	children, patients	148	**0%**	High ROB	Unclear ROB	Unclear ROB	High
**Pakistan**								
Darwish [19]	1983*	patients	43	**9.3%**	High ROB	Unclear ROB	Unclear ROB	Low
Akram [20]	1994	AFI patients	122	**9.8-14.6%**	High ROB	n/a	Low ROB	High
Siddiqui [21]	1999-2001	AFI patients	341	**15.8%**	High ROB	n/a	Low ROB	High
Tariq [22]	2003	suspected dengue	52	**73%**	High ROB	n/a	Unclear ROB	Low
Jamil [23]	2005	suspected dengue	106	**36.8%**	High ROB	n/a	Unclear ROB	High
Khan [24]	2006	suspected dengue	83	**50.7-87.9%**	Low ROB	n/a	Low ROB	Low
Khan [25]	2006	suspected dengue	250	**23.2-74%**	Low ROB	n/a	Low ROB	High

Koo [26]	2006-11	suspected dengue	200	**47%**	Low ROB	n/a	Unclear ROB	High
Khan [27]	2006-07	suspected dengue	50	**40%**	High ROB	n/a	Low ROB	Low
Khan [28]	2006-07	suspected dengue	15,040	**26.3%**	High ROB	n/a	Low ROB	High
Abbasi [29]	2007-08	suspected dengue	114	**69.6%**	High ROB	n/a	Low ROB	High
Tahir [30]	2008	suspected dengue	3215	**54.9%**	High ROB	n/a	Low ROB	High
Murad [31]	2008	suspected dengue	70	**17.1%**	High ROB	n/a	Unclear ROB	Low
Mahmood [32]	2008	suspected dengue	200	**39.5%**	High ROB	n/a	Low ROB	High
		suspected dengue	341	**48.7%**	High ROB	n/a	Low ROB	High
Kidwai [33]	2008-09	suspected dengue	599	**41.9-83.2%**	High ROB	n/a	Low ROB	High
Zafar [34]	2009	general population	96	**19.8%**	High ROB	Low ROB	Unclear ROB	Low
Zafar [35]	2009	general population	244	**28.8%**	High ROB	Unclear ROB	Unclear ROB	High
Qureshi [36]	2010-12	suspected dengue	162	**9.9%**	High ROB	n/a	Low ROB	High
Khan [37]	2010	suspected dengue	125	**54.4%**	High ROB	n/a	Low ROB	High
Hasan [38]	2010	suspected dengue	259	**34.8%**	High ROB	n/a	Unclear ROB	High
Umar [39]	2010	suspected dengue	500	**6.8%**	High ROB	n/a	Low ROB	High
Jameel [40]	2010	suspected dengue	341	**48.7%**	High ROB	n/a	Low ROB	High
Naeem [41]	2011	suspected dengue	79	**25.3%**	High ROB	n/a	Unclear ROB	Low
Ahmed [42]	2011	suspected dengue	640	**43.9%**	High ROB	n/a	Unclear ROB	High
Ijaz [43]	2011	suspected dengue	5,274	**49%**	High ROB	n/a	Unclear ROB	High
Rashid [44]	2011	suspected dengue	254	**36.6%**	High ROB	n/a	Low ROB	High
Khan [45]	2011	suspected dengue	50	**60-72%**	Low ROB	n/a	Low ROB	Low
Hasan [46]	2007-13	suspected Crimean-Congo Hemorrhagic Fever	168	**33.9%**	Low ROB	n/a	Low ROB	High
Ali [47]	2011	suspected dengue	612	**20.2%**	High ROB	n/a	Unclear ROB	High
Hisam [48]	2012	AFI patients	500	**3.2%**	High ROB	n/a	Low ROB	High
Assir[49]	2012	suspected dengue	85	**43.5%**	Low ROB	n/a	Unclear ROB	Low
**Saudi Arabia**								
Fakeeh [50]	1994-99	suspected dengue	985	**16.2-31.9%**	Low ROB	n/a	Low ROB	High
Fakeeh [51]	1994-2002	suspected dengue	1020	**10.8-50.5%**	Low ROB	n/a	Low ROB	High
Khan [52]	2004	suspected dengue	136	**32.4-58.8%**	Low ROB	n/a	Unclear ROB	High
Ayyub [53]	2004-05	suspected dengue	80	**48.8%**	High ROB	n/a	Unclear ROB	Low
Shahin [54]	2006-08	suspected dengue	159	**100%**	Low ROB	n/a	Low ROB	High
Said [55]	2006	suspected dengue	525	**19.2%**	High ROB	n/a	Low ROB	High
Memish [56]	2010	adult military	1024	**0.1%**	High ROB	Unclear ROB	Unclear ROB	High
Gamil [57]	2010-11	suspected dengue	553	**47.7%**	Unclear ROB	n/a	Unclear ROB	High
Al-Azraqi [58]	2013	patients	268	**26.5%**	High ROB	Low ROB	Unclear ROB	High
		patients	697	**33.7%**	High ROB	Low ROB	Unclear ROB	High
Ashshi [59]	2014	blood donors	100	**1-7%**	High ROB	High ROB	Low ROB	High
**Somalia**								
Botros [60]	1987	AFI patients	38	**14.2-60.7%**	Low ROB	n/a	Unclear ROB	Low
Kanesa-thasan [61]	1993	AFI patients	84	**17.8%**	Low ROB	n/a	Unclear ROB	Low
Sharp [62]	1992-93	AFI military soldiers	129	**34.9%**	Low ROB	n/a	Unclear ROB	
		adult military	494	**7.7%**	High ROB	High ROB	Low ROB	High
Nur [63]	1995	children	23	**0%**	High ROB	High ROB	Unclear ROB	Low
		AFI patients	46	**0%**	High ROB	n/a	Unclear ROB	Low
Kyobe Bosa [64]	2011	AFI patients	134	**62-80%**	Low ROB	n/a	Low ROB	High
**Sudan**								
Omer [65]	1976	general population	109	**17.4-27.5%**	Low ROB	High ROB	Unclear ROB	High

Hyams [66]	1984	AFI patients	100	**21%**	Low ROB	n/a	Unclear ROB	High
Woodruff [67]	1986	AFI patients	130	**40.0%**	High ROB	n/a	Unclear ROB	High
McCarthy [68]	1988	patients	100	**49%**	High ROB	High ROB	Unclear ROB	High
		AFI patients	196	**48%**	High ROB	n/a	Low ROB	High
Watts [69]	1989	AFI patients	185	**24.0%**	High ROB	n/a	Unclear ROB	High
Ibrahim [70]	1997-99	suspected measles	188	**3.2%**	High ROB	n/a	Low ROB	High
Malik [71]	2004-05	Hospitals; suspected dengue	40	**90.0%**	Low ROB	n/a	Low ROB	Low
Gould [72]	2005	AFI patients	34	**5.9%** **	High ROB	n/a	Low	Low
Farnon [73]	2005	general population	87	**1.1%**	Low ROB	Low ROB	Unclear ROB	Low
Seidahmed [74]	2008-09	general population	791	**5.2%**	High ROB	Low ROB	Low ROB	High
Adam [75]	2008-09	pregnant women	10,820	**0.7%**	High ROB	High ROB	Low ROB	High
Himatt [76]	2011	general population	489	**0.6-9.4%**	High ROB	Low ROB	Low ROB	High
Abdalla [77]	2012	AFI patients	60	**11.7%**	High ROB	n/a	Low ROB	Low
Elduma [78]	2012	pregnant women with AFI	39	**12.8%**	Low ROB	n/a	Low ROB	Low
Soghaier [79]	2014	general population	600	**27.7%**	High ROB	Low ROB	Low ROB	High
**Turkey**								
Ari [80]	1971	general population	270	**0%**	High ROB	High ROB	Unclear ROB	High
Radda [81]	1973*	general population	270	**0.3%**	High ROB	High ROB	Unclear ROB	High
		general population	90	**0%**	High ROB	High ROB	Unclear ROB	Low
		general population	95	**0%**	High ROB	High ROB	Unclear ROB	Low
Ergunay [82]	2010	blood donors	2435	**0.9%**	High ROB	Low ROB	Unclear ROB	High
Tezcan [83]	2010-11	blood donors	920	**0.9-16.6%**	High ROB	Low ROB	Unclear ROB	High
**Yemen**								
Bin Ghouth [84]	2011	suspected dengue	982	**50.6-64.1%**	Low ROB	n/a	Low ROB	High
Malik [85]	2010-11	AFI patients	136	**8.1-87.5%**	High ROB	n/a	Low ROB	High
Madani [86]	2010	suspected viral hemorrhagic fever	207	**0.9-78.7%**	Low ROB	n/a	Low ROB	High
Rezza [87]	2012	suspected dengue	400	**13.8%**	Low ROB	n/a	Unclear ROB	High
Qassem [88]	2013	suspected dengue or West Nile fever	42	**19.0%**	High ROB	n/a	Unclear ROB	Low

* Since the populations of acute febrile illness and suspected dengue infection are defined as populations presenting to a health facility with acute infection, no population-based sampling is needed to capture these populations and they are denoted ‘n/a’ in the sampling column.

Abbreviation: AFI, acute febrile illness

References

1. Elyan DS, Moustafa L, Noormal B, Jacobs JS, Aziz MA, Hassan KS, et al. Serological evidence of Flaviviruses infection among acute febrile illness patients in Afghanistan. J Infect Dev Ctries. 2014;8(9):1176-80. doi: 10.3855/jidc.4183. PMID: 25212082.
2. Salah S, Fox E, Abbatte EA, Constantine NT, Asselin P, Soliman AK. A negative human serosurvey of haemorrhagic fever viruses in Djibouti. Annales de l'Institut Pasteur Virology. 1988;139(4):439-42. PMID: 1989024043.
3. Rodier GR, Gubler DJ, Cope SE, Cropp CB, Soliman AK, Polycarpe D, et al. Epidemic dengue 2 in the city of Djibouti 1991-1992. Transactions of the Royal Society of Tropical Medicine and Hygiene. 1996;90(3):237-40. doi: http://dx.doi.org/10.1016/S0035-9203%2896%2990228-X. PMID: 1996194667.
4. Faulde MK, Spiesberger M, Abbas B. Sentinel site-enhanced near-real time surveillance documenting West Nile virus circulation in two Culex mosquito species indicating different transmission characteristics, Djibouti City, Djibouti. Journal of the Egyptian Society of Parasitology. 2012;42(2):461-74. PMID: 23214223.
5. Andayi F, Charrel RN, Kieffer A, Richet H, Pastorino B, Leparc-Goffart I, et al. A sero-epidemiological study of arboviral fevers in Djibouti, horn of Africa. PLoS Negl Trop Dis. 2014;8(12):e3299. doi: 10.1371/journal.pntd.0003299. PMID: 25502692.

6. Mohammed YS, Sekeyova M, Gresikova M, el-Dawala K. Studies on arboviruses in Egypt. I. Hemagglutination-inhibition antibodies against arboviruses in human population of Alexandria and Abyss areas. The Indian journal of medical research. 1968;56(4):381-5. PMID: 5687693.
7. Mohammed YS, Gresikova M, Adamyova K, Ragib AHe-DK. Studies on arboviruses in Egypt. II. Contribution of arboviruses to the aetiology of undiagnosed fever among children. The Journal of hygiene. 1970;68(3):491-5. PMID: 5272347.
8. Darwish MA, Ibrahim AH. Prevalence of antibodies to arboviruses in Egypt. Results of a serologic survey among 1,113 university students. American Journal of Tropical Medicine and Hygiene. 1975;24(6 I):981-5. PMID: 0976189164.
9. Saidi S. Survey of antibodies to arboviruses in human population of Iran. Pahlavi Medical Journal. 1971;2(3):485-90. PMID: 0008837992.
10. Saidi S. Viral antibodies in preschool children from the Caspian area, Iran. Iranian Journal of Public Health. 1974;3(2):83-91. PMID: 0975149818.
11. Chinikar S, Ghiasi SM, Shah-Hosseini N, Mostafavi E, Moradi M, Khakifirouz S, et al. Preliminary study of dengue virus infection in Iran. Travel Medicine and Infectious Disease. 2013;11(3):166-9. doi: http://dx.doi.org/10.1016/j.tmaid.2012.10.001. PMID: 2013353214.
12. Aghaie A, Aaskov J, Chinikar S, Niedrig M, Banazadeh S, Mohammadpour HK. Frequency of dengue virus infection in blood donors in Sistan and Baluchestan province in Iran. Transfusion and Apheresis Science. 2014;50(1):59-62. doi: http://dx.doi.org/10.1016/j.transci.2013.07.034. PMID: 2014206312.
13. Ibrahim SH, Darwish MA, Wahdan MH, El Ghoroury AAA. Survey for antibodies against group B arboviruses in man in Kuwait. Journal of the Egyptian Public Health Association. 1974;49(2):77-95. PMID: 0975155283.
14. Al-Nakib W, Lloyd G, El-Mekki A, Platt G, Beeson A, Southee T. Preliminary report on arbovirus-antibody prevalence among patients in Kuwait: evidence of Congo/Crimean virus infection. Transactions of the Royal Society of Tropical Medicine and Hygiene. 1984;78(4):474-6. PMID: 6435292.
15. Pacsa A, Mustafa AS, Chaturvedi UC. Study of dengue virus infection in Kuwait. Dengue Bulletin. 2002;26(pp 113-117). PMID: 2003387961.
16. Garabedian GA, Matossian RM, Musalli MN. Serologic evidence of arbovirus infection in Lebanon. Le Journal medical libanais. 1971;The Lebanese medical journal. 24(4):339-50. PMID: 5149804.
17. Hatem J. The role of the laboratory in the surveillance of viral diseases in Lebanon. Journal Medical Libanais. 1972;25(3):151-65. PMID: 0007410031.
18. Darwish MA, Ibrahim AH. A serological survey on group A and B arbovirus antibodies in Libya. Journal of the Egyptian Public Health Association. 1974;49(1):20-6. PMID: 0975093643.
19. Darwish MA, Hoogstraal H, Roberts TJ, Ahmed IP, Omar F. A sero-epidemiological survey for certain arboviruses (Togaviridae) in Pakistan. Transactions of the Royal Society of Tropical Medicine and Hygiene. 1983;77(4):442-5. PMID: 6314612.
20. Akram DS, Igarashi A, Takasu T. Dengue virus infection among children with undifferentiated fever in Karachi. Indian journal of pediatrics. 1998;65(5):735-40. PMID: 10773930.
21. Siddiqui FJ, Haider SR, Bhutta ZA. Endemic Dengue Fever: A seldom recognized hazard for Pakistani children. Journal of Infection in Developing Countries. 2009;3(4):306-12. PMID: 2009318550.
22. Tariq W KS, Hussain A, Bhani E. Outbreak of dengue fever in Mangla and Mirpur area. Pak J Pathol. 2006;17(3):122.
23. Jamil B, Hasan R, Zafar A, Bewley K, Chamberlain J, Mioulet V, et al. Dengue virus serotype 3, Karachi, Pakistan. Emerg Infect Dis. 2007;13(1):182-3.
24. Khan E, Mehraj V, Nasir A, Khan NA, Billoo B, Moatter T, et al. Evaluation of two ELISA Assay Kits against RT-PCR for diagnosis of Dengue Virus Infection in a Hospital Setting in Karachi, Pakistan. Journal of the Pakistan Medical Association. 2009;59(6):390-4. PMID: 2009286971.
25. Khan E, Hasan R, Mehraj V, Nasir A, Siddiqui J, Hewson R. Co-circulations of two genotypes of dengue virus in 2006 out-break of dengue hemorrhagic fever in Karachi, Pakistan. Journal of Clinical Virology. 2008;43(2):176-9. doi: http://dx.doi.org/10.1016/j.jcv.2008.06.003. PMID: 2008418044.
26. Koo C, Nasir A, Hapuarachchi HC, Lee KS, Hasan Z, Ng LC, et al. Evolution and heterogeneity of multiple serotypes of Dengue virus in Pakistan, 2006-2011. Virology Journal. 2013. doi: http://dx.doi.org/10.1186/1743-422X-10-275. PMID: 2013586909.
27. Khan AH, Hayat AS, Masood N, Solangi NM, Shaikh TZ. Frequency and clinical presentation of dengue fever at tertiary care hospital of Hyderabad/Jamshoro. Journal of the Liaquat University of Medical and Health Sciences. 2010;9(2):88-94. PMID: 2010602294.
28. Khan E, Kisat M, Khan N, Nasir A, Ayub S, Hasan R. Demographic and clinical features of dengue fever in Pakistan from 2003-2007: A retrospective cross- sectional study. PLoS ONE. 2010;5(9):1-7. doi: http://dx.doi.org/10.1371/journal.pone.0012505. PMID: 2010592334.
29. Abbasi A, Butt N, Sheikh QH, Bhutto AR, Munir SM, Ahmed SM. Clinical features, diagnostic techniques and management of dual dengue and Malaria infection. Journal of the College of Physicians and Surgeons Pakistan. 2009;19(1):25-9. PMID: 2009145621.
30. Tahir Z HS, Chaudhry A. Spatial and seasonal varation of dengue fever in Lahore 2008. Biomedica. 2010;26(Jul-Dec.):166.

31. Murad H, Asahar RJ, Zaheen M, Shawali R. Outbreak investigation of Dengue fever in Sundia, Chakaiser, Shangla, Pakistan-2008. Journal of Ayub Medical College, Abbottabad : JAMC. 2014;26(4):571-6. PMID: 25672190.
32. Mahmood K AH, Tahir M. Incidence of dengue haemorrhagic fever in local population of Lahore, Pakistan. Biomedica. 2009;25(Jul.-Dec.):93-6.
33. Kidwai AA, Jamal Q, Saher, Mehrunnisa, Farooqi FU, Saleem U. Serodiagnosis of dengue infection using rapid immunochromatography test in patients with probable dengue infection. J Pak Med Assoc. 2010;60(11):936-9.
34. Zafar H, Hayyat A, Akhtar N, Rizwan SF. Prevalence of undifferentiated fever in adults of Rawalpindi having primary dengue fever. JPMA The Journal of the Pakistan Medical Association. 2013;63(6):770-1. PMID: 23901683.
35. Zafar H, Hayyat A, Akhtar N. Incidence of primary dengue viral infection in healthy adults of Rawalpindi, Pakistan. Journal of the Pakistan Medical Association. 2011;61(10):1030-31. PMID: 2011534565.
36. Qureshi KA LA, Samoo AH. Screening for dengue virus infection at GMMMC hospital, Sukkur. Medical Forum Monthly. 2013;24(4):6-8.
37. Khan H KQ, Khan BA, Arif M, Raza AAH. Retrospective analysis of 68 cases of dengue fever. Pak J Med Res. 2012;51(1):18.
38. Hasan SR, Riaz M, Jafri FA. Characteristics and outcome of dengue infection; clinical perspective from a secondary care hospital of Karachi. Pakistan Journal of Medical Sciences. 2013;29(1):115-8. doi: http://dx.doi.org/http://dx.doi.org/10.12669/pjms.291.2742. PMID: 2013068279.
39. Umar S, Ashraf O, Umar M. Characteristics of febrile thrombocytopenia during dengue epidemic 2010 in Rawalpindi, Pakistan. International Journal of Infectious Diseases. 2011;(15):S114-S115. doi: http://dx.doi.org/10.1016/S1201-9712%2811%2960399-8. PMID: 70496826.
40. Jameel T MK, Ghulam Choudhry N, Afzal NP, Paul RF. Changing hematologic parameters in dengue viral infections. J Ayub med Coll-Abbotabad-Pak. 2012;24(1):3.
41. Naeem M SA, Batool S, Rubab S, Saba T, Riaz T, Mahmood A. Dengue fever; a clinical experience Professional Med J. 2014;23(2):243-46.
42. Ahmed S, Mohammad WW, Hamid F, Akhter A, Afzal RK, Mahmood A. The 2011 dengue haemorrhagic fever outbreak in lahore - an account of clinical parameters and pattern of haemorrhagic complications. Journal of the College of Physicians and Surgeons Pakistan. 2013;23(7):463-7. PMID: 2013472360.
43. Ijaz T, Ijaz S, Aslam S, Ahmad BM, Raja SA. A laboratory based study of dengue epidemic in the city of Lahore during year 2011. International Journal of Infectious Diseases. 2014;Conference:16th International Congress on Infectious Diseases. 2014;(21):136. doi: http://dx.doi.org/10.1016/j.ijid.2014.03.708. PMID: 71634046.
44. Rashid A KH, Nadeem UR. Dengue Hemorrhagic Fever / dengue shock syndrome. Professional Med J. 2012;19(5):661.
45. Khan MA, Ellis EM, Tissera HA, Alvi MY, Rahman FF, Masud F, et al. Emergence and Diversification of Dengue 2 Cosmopolitan Genotype in Pakistan, 2011. PLoS ONE. 2013;8(3). doi: http://dx.doi.org/10.1371/journal.pone.0056391. PMID: 2013153148.
46. Hasan Z, Atkinson B, Jamil B, Samreen A, Altaf L, Hewson R. Short report: Diagnostic testing for hemorrhagic fevers in Pakistan: 2007-2013. American Journal of Tropical Medicine and Hygiene. 1243;91(6):1243-6. doi: http://dx.doi.org/10.4269/ajtmh.14-0383. PMID: 2014984911.
47. Ali A, Rehman HU, Nisar M, Rafique S, Ali S, Hussain A, et al. Seroepidemiology of dengue fever in Khyber Pakhtunkhawa, Pakistan. International Journal of Infectious Diseases. 2013;17(7):e518-e23. doi: http://dx.doi.org/10.1016/j.ijid.2013.01.007. PMID: 2013302716.
48. Hisam A, Mahmood ur R, Khan MB, Kadir E, Azam N. Frequency of co-existence of dengue and malaria in patients presenting with acute febrile illness. JPMA The Journal of the Pakistan Medical Association. 2014;64(3):247-51. PMID: 24864593.
49. Assir MZK, Masood MA, Ahmad HI. Concurrent dengue and malaria infection in Lahore, Pakistan during the 2012 dengue outbreak. International Journal of Infectious Diseases. 2014;18(1):41-6. doi: http://dx.doi.org/10.1016/j.ijid.2013.09.007. PMID: 2014008756.
50. Fakeeh M, Zaki AM. Virologic and serologic surveillance for dengue fever in Jeddah, Saudi Arabia, 1994-1999. American Journal of Tropical Medicine and Hygiene. 2001;65(6):764-7. PMID: 2002021757.
51. Fakeeh M, Zaki AM. Dengue in Jeddah, Saudi Arabia, 1994-2002. Dengue Bulletin. 2003;(27):13-18. PMID: 2004280253.
52. Khan NA, Azhar EI, El-Fiky S, Madani HH, Abuljadial MA, Ashshi AM, et al. Clinical profile and outcome of hospitalized patients during first outbreak of dengue in Makkah, Saudi Arabia. Acta Tropica. 2008;105(1):39-44. doi: http://dx.doi.org/10.1016/j.actatropica.2007.09.005. PMID: 2007609584.
53. Ayyub M, Khazindar AM, Lubbad EH, Barlas S, Alfi AY, Al-Ukayli S. Characteristics of dengue fever in a large public hospital, Jeddah, Saudi Arabia. Journal of Ayub Medical College, Abbottabad. 2006;18(2):9-13. PMID: 16977805.
54. Shahin W, Nassar A, Kalkattawi M, Bokhari H. Dengue fever in a tertiary hospital in Makkah, Saudi Arabia. Dengue Bulletin. 2009;33(1):34-44. PMID: 2011641978.

55. Said SM EK, Alyan Z. Benign acute myositis in association with acute dengue viruses' infections. Egypt J Neurol Psychiatry Neurosurg. 2008;45(1):193.
56. Memish ZA, Albarrak A, Almazroa MA, Al-Omar I, Alhakeem R, Assiri A, et al. Seroprevalence of Alkhurma and other hemorrhagic fever viruses, Saudi Arabia. Emerging Infectious Diseases. 2011;17(12):2316-8. doi: http://dx.doi.org/http://dx.doi.org/10.3201/eid1712.110658. PMID: 2011666410.
57. Gamil MA, Eisa ZM, Eifan SA, Al-Sum BA. Prevalence of dengue fever in Jizan area, Saudi Arabia. Journal of Pure and Applied Microbiology. 2014;8(1):225-31. PMID: 2014242333.
58. Al-Azraqi TA, El Mekki AA, Mahfouzc AA. Seroprevalence of dengue virus infection in Aseer and Jizan regions, Southwestern Saudi Arabia. Transactions of the Royal Society of Tropical Medicine and Hygiene. 2013;107(6):368-71. doi: http://dx.doi.org/10.1093/trstmh/trt022. PMID: 2013533560.
59. Ashshi AM. Serodetection of Dengue virus and its antibodies among blood donors in the western region of Saudi Arabia: a preliminary study. Blood Transfus. 2015;13(1):135-8. doi: 10.2450/2014.0134-14. PMID: 25369603.
60. Botros BAM, Watts DM, Soliman AK, Salib AW, Moussa MI, Mursal H, et al. Serological evidence of dengue fever among refugees, Hargeysa, Somalia. Journal of Medical Virology. 1989;29(2):79-81. PMID: 1989282288.
61. Kanesa-thasan N, Iacono-Connors L, Magill A, Smoak B, Vaughn D, Dubois D, et al. Dengue serotypes 2 and 3 in US forces in Somalia. Lancet. 1994;343(8898):678.
62. Sharp TW, Wallace MR, Hayes CG, Sanchez JL, DeFraites RF, Arthur RR, et al. Dengue fever in U.S. troops during Operation Restore Hope, Somalia, 1992-1993. Am J Trop Med Hyg. 1995;53(1):89-94.
63. Nur YA, Groen J, Yusuf MA, Osterhaus ADME. IgM antibodies in hospitalized children with febrile illness during an inter-epidemic period of measles, in Somalia. Journal of Clinical Virology. 1999;12(1):21-5. doi: http://dx.doi.org/10.1016/S1386-6532%2898%2900002-X. PMID: 1999063004.
64. Kyobe Bosa H, Montgomery JM, Kimuli I, Lutwama JJ. Dengue fever outbreak in Mogadishu, Somalia 2011: Co-circulation of three dengue virus serotypes. International Journal of Infectious Diseases. 2014;(21):3. doi: http://dx.doi.org/10.1016/j.ijid.2014.03.412. PMID: 71633760.
65. Omer AHS, McLaren ML, Johnson BK. A seroepidemiological survey in the Gezira, Sudan, with special reference to arboviruses. Journal of Tropical Medicine and Hygiene. 1981;84(2):63-6. PMID: 1981213114.
66. Hyams KC, Oldfield EC, McNair Scott R. Evaluation of febrile patients in Port Sudan, Sudan: Isolation of dengue virus. American Journal of Tropical Medicine and Hygiene. 1986;35(4):860-5. PMID: 1986196316.
67. Woodruff PWR, Morrill JC, Burans JP, Hyams KC, Woody JN. A study of viral and rickettsial exposure and causes of fever in Juba, southern Sudan. Transactions of the Royal Society of Tropical Medicine and Hygiene. 1988;82(5):761-6. doi: http://dx.doi.org/10.1016/0035-9203%2888%2990229-5. PMID: 1988257341.
68. McCarthy MC, Haberberger RL, Salib AW, Soliman BA, El-Tigani A, Khalid IO, et al. Evaluation of arthropod-borne viruses and other infectious disease pathogens as the causes of febrile illnesses in the Khartoum province of Sudan. Journal of Medical Virology. 1996;48(2):141-6. doi: http://dx.doi.org/10.1002/%28SICI%291096-9071%28199602%2948:2%3C141::AID-JMV4%3E3.0.CO;2-9. PMID: 1996056198.
69. Watts DM, El-Tigani A, Botros BAM, Salib AW, Olson JG, McCarthy M, et al. Arthropod-borne viral infections associated with a fever outbreak in the Northern Province of Sudan. Journal of Tropical Medicine and Hygiene. 1994;97(4):228-30. PMID: 1994258772.
70. Ibrahim SA, Mustafa OM, Mukhtar MM, Saleh EA, El Mubarak HS, Abdallah A, et al. Measles in suburban Khartoum: An epidemiological and clinical study. Tropical Medicine and International Health. 2002;7(5):442-9. doi: http://dx.doi.org/10.1046/j.1365-3156.2002.00884.x. PMID: 2002183345.
71. Malik A, Earhart K, Mohareb E, Saad M, Saeed M, Ageep A, et al. Dengue hemorrhagic fever outbreak in children in Port Sudan. Journal of Infection and Public Health. 2011;4(1):1-6. doi: http://dx.doi.org/10.1016/j.jiph.2010.08.001. PMID: 2011100176.
72. Gould LH, Osman MS, Farnon EC, Griffith KS, Godsey MS, Karch S, et al. An outbreak of yellow fever with concurrent chikungunya virus transmission in South Kordofan, Sudan, 2005. Transactions of the Royal Society of Tropical Medicine and Hygiene. 2008;102(12):1247-54. doi: http://dx.doi.org/10.1016/j.trstmh.2008.04.014. PMID: 2008503238.
73. Farnon EC, Gould LH, Griffith KS, Osman MS, El Kholy A, Brair ME, et al. Household-Based Sero-Epidemiologic Survey after a Yellow Fever Epidemic, Sudan, 2005. American Journal of Tropical Medicine and Hygiene. 2010;82(6):1146-52. doi: http://dx.doi.org/10.4269/ajtmh.2010.09-0105. PMID: 2010347047.
74. Seidahmed OME, Hassan SA, Soghaier MA, Siam HAM, Ahmed FTA, Elkarsany MM, et al. Spatial and Temporal Patterns of Dengue Transmission along a Red Sea Coastline: A Longitudinal Entomological and Serological Survey in Port Sudan City. PLoS Neglected Tropical Diseases. 2012;6(9). doi: http://dx.doi.org/10.1371/journal.pntd.0001821. PMID: 2012579248.

75. Adam I, Jumaa AM, Elbashir HM, Karsany MS. Maternal and perinatal outcomes of dengue in PortSudan, Eastern Sudan. Virology Journal. 2010;7(153). doi: http://dx.doi.org/10.1186/1743-422X-7-153. PMID: 2010416905.
76. Himatt S, Osman KE, Okoued SI, Seidahmed OE, Beatty ME, Soghaier MA, et al. Sero-prevalence of dengue infections in the Kassala state in the eastern part of the Sudan in 2011. J Infect Public Health. 2015. doi: 10.1016/j.jiph.2015.04.023. PMID: 25975993.
77. Abdalla TM, Karsany MS, Ali AA. Correlation of measles and dengue infection in Kassala, Eastern Sudan. J Med Virol. 2014. doi: 10.1002/jmv.24001. PMID: 24980486.
78. Elduma AH, Osman WM. Dengue and hepatitis E virus infection in pregnant women in Eastern Sudan, a challenge for diagnosis in an endemic area. Pan Afr Med J. 2014;19:391. doi: 10.11604/pamj.2014.19.391.5439. PMID: 25995787.
79. Soghaier MA, Mahmood SF, Pasha O, Azam SI, Karsani MM, Elmangory MM, et al. Factors associated with dengue fever IgG sero-prevalence in South Kordofan State, Sudan, in 2012: Reporting prevalence ratios. Journal of Infection and Public Health. 2014;7(1):54-61. doi: http://dx.doi.org/10.1016/j.jiph.2013.07.008. PMID: 2014063379.
80. Ari A. The prevalence and ecology of arboviruses in Turkey. Turkiyede arboviruslarin faaliyeti ve ekolojisi uzerinde incelemeler. Turk hijiyen ve tecrubi biyoloji dergisi. 1972;32(2):134-43. PMID: 4668350.
81. Radda A. Studies on the activity and ecology of arboviruses in Turkey. Untersuchungen Uber Die Aktivitat Und Okologie Von Arboviren in Der Turkei. ZblBaktReihe A. 1973;225(1):19-26. PMID: 0974127896.
82. Ergunay K, Saygan MB, Aydogan S, Litzba N, Niedrig M, Pinar A, et al. Investigation of dengue virus and yellow fever virus seropositivities in blood donors from central/Northern Anatolia, Turkey. Mikrobiyoloji Bulteni. 2010;44(3):415-24. PMID: 21063991.
83. Tezcan S, Kizildamar S, Ulger M, Aslan G, Tiftik N, Ozkul A, et al. Flavivirus seroepidemiology in blood donors in Mersin province, Turkey. Mikrobiyoloji bulteni. 2014;48(4):606-17. PMID: 25492656.
84. Bin Ghouth AS, Amarasinghe A, Letson GW. Dengue outbreak in Hadramout, Yemen, 2010: an epidemiological perspective. Am J Trop Med Hyg. 2012;86(6):1072-6. doi: 10.4269/ajtmh.2012.11-0723.
85. Malik MR, Mnzava A, Mohareb E, Zayed A, Al Kohlani A, Thabet AAK, et al. Chikungunya outbreak in Al-Hudaydah, Yemen, 2011: Epidemiological characterization and key lessons learned for early detection and control. Journal of Epidemiology and Global Health. 2014;4(3):203-11. doi: http://dx.doi.org/10.1016/j.jegh.2014.01.004. PMID: 2014527198.
86. Madani TA, Abuelzein ETME, Al-Bar HMS, Azhar EI, Kao M, Alshoeb HO, et al. Outbreak of viral hemorrhagic fever caused by dengue virus type 3 in Al-Mukalla, Yemen. BMC Infectious Diseases. 2013;13(1). doi: http://dx.doi.org/10.1186/1471-2334-13-136. PMID: 2013189579.
87. Rezza G, El-Sawaf G, Faggioni G, Vescio F, Al Ameri R, De Santis R, et al. Co-circulation of Dengue and Chikungunya Viruses, Al Hudaydah, Yemen, 2012. Emerg Infect Dis. 2014;20(8):1351-4. doi: 10.3201/eid2008.131615.
88. Qassem MAM, Jaawal AAT. Dengue fever or West Nile virus outbreak? Yemen 2013. International Journal of Infectious Diseases. 2014;Conference:16th International Congress on Infectious Diseases, ICID 2014 Cape Town South Africa. Conference Publication: 21 (pp 457). doi: http://dx.doi.org/10.1016/j.ijid.2014.03.1364. PMID: 71634698.
