# Supplementary material for: Dengue in the Middle East and North Africa: A Systematic Review
Source: PLoS Negl Trop Dis. 2016 Dec 7;10(12):e0005194. doi: 10.1371/journal.pntd.0005194 (PMC5142774; doi:10.1371/journal.pntd.0005194)
Supplement: S2 Table — (PDF) [file pntd.0005194.s004.pdf]

S2 Table. Summary of reported outbreaks and sentinel cases for dengue virus in the Middle East and North Africa.

Country, Year	City or Governorate	Description	Ref.
**Dengue virus**			
**Djibouti**			
1991-92	Djibouti City	12,000 estimated cases; DEN-2	[1, 2]
2010-11	Djibouti City	4 cases in foreign military personnel; DEN-1,2,3	[3]
**Egypt**			
1799	Cairo, Alexandria	unconfirmed outbreak	[4]
1871	Port Said	unconfirmed outbreak	[4]
1880	Cairo	unconfirmed outbreak	[4]
1889	Nile Delta	unconfirmed outbreak	[4]
1906	Port Said, Suez Canal	unconfirmed outbreak	[5]
1927	Countrywide	unconfirmed outbreak	[5, 6]
1937	n/a	n/a	[4]
2010	Port Ghalib	2 cases in travelers returning from Egypt	[4]
2015	Assiut	253 cases; DEN-1	[7]
**Iraq**			
1944	n/s	unconfirmed outbreak based on clinical diagnosis	[8, 9]
**Lebanon**			
1945-46	Beirut	>100,000 suspected cases	[10]
**Pakistan**			
1994-1995	Karachi	first confirmed outbreak of DENV in Pakistan; 145 reported cases; DEN-1,2	[11-16]
2003	n/a	DEN-1,2	[16, 17]
2005-06	Karachi	3,640 cases; DEN-3	[13, 14, 16, 18]
2006	Multiple	1,931 confirmed cases; DEN-2,3	[16, 19]
2007	Multiple	1,226 confirmed cases; DEN-2,3	[16, 19]
2008	Multiple	2,469 confirmed cases; DEN-2,3,4	[19, 20]
2009	Lahore, multiple	1,085 confirmed cases; DEN-2,3,4	[19]
2010	Multiple	11,024 confirmed cases	[19] [21]
2011	Punjab, multiple	21,580 confirmed cases; DEN-2	[19, 22]
2013	Khyber Pakhtunkhwa	9,024 reported cases	[23, 24]
	Sindh	>6,000 reported cases	[23]
2014	Lahore	1,799 reported cases	[25]
**Saudi Arabia**			
1994	Jeddah	First detection of autochthonous transmission of DENV in Saudi Arabia;	[15, 26]
	Jeddah	469 cases; DEN-2	[15]
		673 suspected cases, 289 confirmed; DEN-1,2	[16, 27]
1995	n/a	136 suspected cases, 6 confirmed; DEN-1,2	[15, 27]
1996	n/a	57 suspected cases, 2 confirmed	[15]
1997	n/a	62 suspected cases, 15 confirmed; DEN-3	[15, 27]
1998	n/a	31 suspected cases, 0 confirmed	[15]
1999	n/a	26 suspected cases, 3 confirmed	[15]
2000	n/a	17 suspected cases, 0 confirmed	[15]
2001		7 suspected cases, 0 confirmed	[15]
2004	Jeddah, Makkah	DEN-3	[16]
2005	n/a	32 confirmed cases; DEN-3	[15, 16]
2006	n/a	DEN-1	
2006	Jeddah and Makkah	>1,269 cases; DEN-1	[15, 16, 28]
2007	Jeddah and Makkah	200 estimated cases; DEN-3	[16, 28]

2008	Jeddah and Makkah	775 estimated cases; DEN-3	[15, 16, 28]
2009	Jeddah and Makkah	1,500 estimated cases	[28]
2010	Jeddah and Makkah	2,200 estimated cases	[28]
2011	Jeddah and Makkah	2,376 estimated cases	[28]
2012	Jeddah and Makkah	800 estimated cases	[28]
2013	Jeddah and Makkah	4,411 estimated cases	[28]
2006-08	Jeddah	n/a	[29]
2015	Mecca	13 suspected cases	[30]
**Somalia**			
1941	Mogadishu	72 reported cases	[31]
1982-1983	Mogadishu	DEN-2	[16, 32, 33]
1985-87	Hargeysa	DEN-2	[33, 34]
1992-1993	Mogadishu	41 reported cases; DEN-1,2,3	[16, 35, 36]
2005	n/s	n/s	[37]
2011	Mogadishu	DEN-1,2,3	[38, 39]
**Sudan**			
1984-86	Port Sudan	18 reported cases; DEN-1,2,3	[32, 40-42]
1989	Northern Province	DEN-2	[43]
2003	Port Sudan	366 reported cases	[41, 43]
2004	Multiple	127 reported cases; DEN-1,2	[16, 41]
2005	Multiple	233 reported cases; DEN-1,2	[16, 41]
2006	Multiple	7 reported cases; DEN-1,2	[16, 41]
2008	Multiple, Port Sudan	449 reported cases; DEN-1,2	[16, 41, 44, 45]
2009	Multiple, Port Sudan	447 reported cases	[41, 45]
2010	Port Sudan, multiple	4,008 reported cases; DEN-2	[41]
2011	Multiple	257 reported cases	[41]
2012	Multiple	59 reported cases	[41]
2014	Red Sea State	738 reported cases	[41]
2015	Darfur, Kassala, Kordofan	557 suspected cases	[46]
**Yemen**			
1983	Dalah	1 case in traveler returning from Yemen	[47]
1994	Shabwah, Al Hudaydah, Mukkala	DEN-3	[16, 48]
2000	Al Hudaydah	653 suspected cases; DEN-2	[2, 16, 48]
2001-2003	Shabwah	DEN-2	[2, 48]
2004	Al Hudaydah, Shabwah	45 suspected cases; DEN-2	[2, 48]
2005-2006	Shabwah, Hadramout, Al Hudaydah	403 suspected cases; DEN-2,3	[2, 48, 49]
2008	Shabwah, Abyan, Taiz, Mukalla	1,001 reported cases; DEN-3	[2, 16, 49]
2009	n/s	900 cases	[49]
2010	Multiple, Hadramout	5,534 suspected cases; DEN-2,3	[49, 50]
2015	Shabwah, Hadramout, Abyan, Maifaa, Eitg, Taiz, Lahj	>20,000 estimated cases	[51-54]

References

1. Rodier GR, Gubler DJ, Cope SE, Cropp CB, Soliman AK, Polycarpe D, et al. Epidemic dengue 2 in the city of Djibouti 1991-1992. Transactions of the Royal Society of Tropical Medicine and Hygiene. 1996;90(3):237-40. doi: http://dx.doi.org/10.1016/S0035-9203%2896%2990228-X. PMID: 1996194667.
2. World Health Organization Regional Office for the Eastern Mediterranean. Dengue outbreak in Yemen. Weekly Epidemiological Monitor. 2008;1(24). Available www.emro.who.int/surveillance-forecasting-response/weekly-epidemiological-monitor/. Accessed 17 Jan 2016.
3. De Laval F, Dia A, Plumet S, Decam C, Leparc Goffart I, Deparis X. Dengue surveillance in the French armed forces: A dengue sentinel surveillance system in countries without efficient local epidemiological surveillance.

Journal of Travel Medicine. 2013;20(4):259-61. doi: http://dx.doi.org/10.1111/j.1708-8305.2012.00674.x. PMID: 2013420761.
4. World Health Organization Regional Office for the Eastern Mediterranean. Travel-associated dengue fever in Egypt. Weekly Epidemiological Monitor. 2010;3(24). Available www.emro.who.int/surveillance-forecasting-response/weekly-epidemiological-monitor/. Accessed 17 Jan 2016.
5. Kamal H. The 1927 Epidemic of Dengue in Egypt. Br Med J. 1928;1(3521):1104-6. PMID: 20773982.
6. O'Meara FJ. Dengue (Eastern Mediterranean). Trans R Soc Trop Med Hyg. 1956;50(2):181. PMID: 13324927.
7. World Health Organization. Dengue Fever - Egypt 2015. Available from: http://www.who.int/csr/don/12-november-2015-dengue/en/. Accessed 12 Dec 2015.
8. Fleming RF, French JM. Dengue in Iraq. Trans R Soc Trop Med Hyg. 1947;40(6):851-60.
9. Feghali A. Dengue, tertian fever and harara in Lebanon; differential diagnosis; abnormal forms and prevention. La Presse medicale. 1951;59(71):1481. PMID: 14891724.
10. Russell PK, Buescher EL, McCown JM, Ordonez J. Recovery of dengue viruses from patients during epidemics in Puerto Rico and East Pakistan. Am J Trop Med Hyg. 1966;15(4):573-9.
11. World Health Organization Regional Office for the Eastern Mediterranean. Dengue fever in Pakistan: Inten-sity declines, threat remains. 2012;5(1). Available www.emro.who.int/surveillance-forecasting-response/weekly-epidemiological-monitor/. Accessed 17 Jan 2016.
12. Chan YC, Salahuddin NI, Khan J, Tan HC, Seah CLK, Li J, et al. Dengue haemorrhagic fever outbreak in Karachi, Pakistan, 1994. Transactions of the Royal Society of Tropical Medicine and Hygiene. 1995;89(6):619-20. doi: http://dx.doi.org/10.1016/0035-9203%2895%2990412-3. PMID: 1996022132.
13. Raza FA, Rehman SU, Khalid R, Ahmad J, Ashraf S, Iqbal M, et al. Demographic and clinico-epidemiological features of dengue fever in Faisalabad, Pakistan. PLoS ONE. 2014;9(3). doi: http://dx.doi.org/10.1371/journal.pone.0089868. PMID: 2014220022.
14. Chan YC, Salahuddin NI, Khan J, Tan HC, Seah CL, Li J, et al. Dengue haemorrhagic fever outbreak in Karachi, Pakistan, 1994. Trans R Soc Trop Med Hyg. 1995;89(6):619-20.
15. World Health Organization. Dengue: Guidelines for Diagnosis, Treatement, Prevention and Control. Geneva, Switzerland. 2009. Available at: http://www.who.int/tdr/publications/documents/dengue-diagnosis.pdf Accessed 1 September 2016.
16. World Health Organization Regional Office for the Eastern Mediterranean. Multiple outbreaks from DF/DHF in the EMR. 2008;1(30). Available www.emro.who.int/surveillance-forecasting-response/weekly-epidemiological-monitor/. Accessed 17 Jan 2016.
17. Tang JW, Khanani MR, Zubairi AM, Lam WY, Lai F, Hashmi K, et al. A wide spectrum of dengue IgM and PCR positivity post-onset of illness found in a large dengue 3 outbreak in Pakistan. Journal of Medical Virology. 2008;80(12):2113-21. doi: http://dx.doi.org/10.1002/jmv.21290. PMID: 2008582978.
18. Ahmed S, Ali N, Ashraf S, Ilyas M, Tariq WUZ, Chotani RA. Dengue fever outbreak: A clinical management experience. Journal of the College of Physicians and Surgeons Pakistan. 2008;18(1):8-12. PMID: 2008142482.
19. World Health Organization Regional Office for the Eastern Mediterranean. Dengue in Pakistan 2013;6(52). Available www.emro.who.int/surveillance-forecasting-response/weekly-epidemiological-monitor/. Accessed 17 Jan 2016.
20. Haider Z, Ahmad FZ, Mahmood A, Waseem T, Shafiq I, Raza T, et al. Dengue fever in Pakistan: A paradigm shift; Changing epidemiology and clinical patterns. Perspectives in Public Health. 2015;135(6):294-8. doi: http://dx.doi.org/10.1177/1757913915599019. PMID: 2015501013.
21. Murad H, Asahar RJ, Zaheen M, Shawali R. Outbreak investigation of Dengue fever in Sundia, Chakaiser, Shangla, Pakistan-2008. J Ayub Med Coll Abbottabad. 2014;26(4):571-6. PMID: 25672190.
22. Ahmed S, Mohammad WW, Hamid F, Akhter A, Afzal RK, Mahmood A. The 2011 dengue haemorrhagic fever outbreak in lahore - an account of clinical parameters and pattern of haemorrhagic complications. Journal of the College of Physicians and Surgeons Pakistan. 2013;23(7):463-7. PMID: 2013472360.
23. Wasim A, Kazi AN, Hussain SA. 2013: Rise of the deadly dengue fever in Pakistan. J Infect Public Health. 2014;7(5):453-4. doi: 10.1016/j.jiph.2014.05.001. PMID: 24986026.
24. Ali Shah I, Asghar R, Baig M, Ja A. Risk factors identification of Dengue fever outbreak in Mansehra-Sept 2013, Shah IA, Baig MA, Ansari JA, Asghar RJ. Annals of Global Health. 2015;81(1):9. PMID: 72073480.
25. Kraemer MUG, Perkins TA, Cummings DAT, Zakar R, Hay SI, Smith DL, et al. Big city, small world: Density, contact rates, and transmission of dengue across Pakistan. Journal of the Royal Society Interface. 2015;12(111). doi: http://dx.doi.org/10.1098/rsif.2015.0468. PMID: 2015488447.

26. Fakeeh M, Zaki AM. Dengue in Jeddah, Saudi Arabia, 1994-2002. Dengue Bulletin. 2003;27:13-8. PMID: 2004280253.
27. Azhar EI, Hashem AM, El-Kafrawy SA, Abol-Ela S, Abd-Alla AMM, Sohrab SS, et al. Complete genome sequencing and phylogenetic analysis of dengue type 1 virus isolated from Jeddah, Saudi Arabia. Virology Journal. 2015;12(1). doi: http://dx.doi.org/10.1186/s12985-014-0235-7. PMID: 2015179071.
28. Aziz AT, Al-Shami SA, Mahyoub JA, Hatabbi M, Ahmad AH, Rawi CSM. An update on the incidence of dengue gaining strength in Saudi Arabia and current control approaches for its vector mosquito. Parasites and Vectors. 2014;7(1). doi: http://dx.doi.org/10.1186/1756-3305-7-258. PMID: 2014410848.
29. Alzahrani AG, Al Mazroa MA, Alrabeah AM, Ibrahim AM, Mokdad AH, Memish ZA. Geographical distribution and spatio-temporal patterns of dengue cases in Jeddah governorate from 2006-2008. Transactions of the Royal Society of Tropical Medicine and Hygiene. 2013;107(1):23-9. doi: http://dx.doi.org/10.1093/trstmh/trs011. PMID: 2013536559.
30. ProMED-mail. Dengue - Saudi Arabia (MECCA) MOH Alert. ProMED-mail 2015; 9 Dec: 20151209.3846844. <http://www.promedmail.org>. Accessed 17 Jan 2016.
31. McCarthy DD, Bagster Wilson D. Dengue in the East African command. Incidence in relation to Aedes prevalence and some clinical features. Transactions of the Royal Society of Tropical Medicine and Hygiene. 1948;42(1):83-8. doi: http://dx.doi.org/10.1016/0035-9203(48)90352-6. PMID: 0007199567.
32. Saleh AS, Hassan A, Scott RM, Mellick PW, Oldfield EC, 3rd, Podgore JK. Dengue in north-east Africa. Lancet. 1985;2(8448):211-2. PMID: 2862392.
33. Botros BAM, Watts DM, Soliman AK, Salib AW, Moussa MI, Mursal H, et al. Serological evidence of dengue fever among refugees, Hargeysa, Somalia. Journal of Medical Virology. 1989;29(2):79-81. PMID: 1989282288.
34. Rodier GR, Parra JP, Kamil M, Chakib SO, Cope SE. Recurrence and emergence of infectious diseases in Djibouti city. Bulletin of the World Health Organization. 1995;73(6):755-9. PMID: 1996056853.
35. Sharp TW, Wallace MR, Hayes CG, Sanchez JL, DeFraites RF, Arthur RR, et al. Dengue fever in U.S. troops during Operation Restore Hope, Somalia, 1992- 1993. American Journal of Tropical Medicine and Hygiene. 1995;53(1):89-94. PMID: 1995228394.
36. Kanesa-thasan N, Chang GJJ, Smoak BL, Magill A, Burrous MJ, Hoke Jr CH. Molecular and epidemiologic analysis of dengue virus isolates from Somalia. Emerging Infectious Diseases. 1998;4(2):299-303. PMID: 1998227052.
37. Ellis BR, Barrett ADT. The enigma of yellow fever in East Africa. Reviews in Medical Virology. 2008;18(5):331-46. doi: http://dx.doi.org/10.1002/rmv.584. PMID: 2008451571.
38. Ochieng CA. Co-circulation of multiple serotypes of dengue virus in somalia, 2011. American Journal of Tropical Medicine and Hygiene. 2012;(1):30. PMID: 71040730.
39. Kyobe Bosa H, Montgomery JM, Kimuli I, Lutwama JJ. Dengue fever outbreak in Mogadishu, Somalia 2011: Co-circulation of three dengue virus serotypes. International Journal of Infectious Diseases. 2014;(21):3. doi: http://dx.doi.org/10.1016/j.ijid.2014.03.412. PMID: 71633760.
40. World Health Organization Regional Office for the Eastern Mediterranean. Dengue fever in Sudan: cases continue to rise. 2010;3(11). Available www.emro.who.int/surveillance-forecasting-response/weekly-epidemiological-monitor/. Accessed 17 Jan 2016.
41. World Health Organization Regional Office for the Eastern Mediterranean. Dengue fever in Sudan: cases continue to rise. 2014;7(25). Available www.emro.who.int/surveillance-forecasting-response/weekly-epidemiological-monitor/. Accessed 17 Jan 2016.
42. Sharp TW, Wallace MR, Hayes CG, Sanchez JL, DeFraites RF, Arthur RR, et al. Dengue fever in U.S. troops during Operation Restore Hope, Somalia, 1992-1993. Am J Trop Med Hyg. 1995;53(1):89-94.
43. Malik A, Earhart K, Mohareb E, Saad M, Saeed M, Ageep A, et al. Dengue hemorrhagic fever outbreak in children in Port Sudan. Journal of Infection and Public Health. 2011;4(1):1-6. doi: http://dx.doi.org/10.1016/j.jiph.2010.08.001. PMID: 2011100176.
44. Seidahmed OME, Siam HAM, Soghaier MA, Abubakr M, Osman HA, Abd Elrhman LS, et al. Dengue vector control and surveillance during a major outbreak in a coastal Red Sea area in Sudan. Eastern Mediterranean Health Journal. 2012;18(12):1217-24. PMID: 2013300757.
45. Adam I, Jumaa AM, Elbashir HM, Karsany MS. Maternal and perinatal outcomes of dengue in PortSudan, Eastern Sudan. Virology Journal. 2010;7(153). doi: http://dx.doi.org/10.1186/1743-422X-7-153. PMID: 2010416905.
46. ProMED-mail. Dengue - Sudan (02): New Areas, fatal, MOH. ProMED-mail. 2015; 12 Dec: 20151212.3854435. <http://www.promedmail.org>. Accessed 17 Jan 2016.

47. Jimenez-Lucho VE, Fisher EJ, Saravolatz LD. Dengue with hemorrhagic manifestations: An imported case from the Middle East. American Journal of Tropical Medicine and Hygiene. 1984;33(4):650-3. PMID: 1984178309.
48. Ghouth ASB, Amarasinghe A, Letson GW. Dengue outbreak in Hadramout, Yemen, 2010: An epidemiological perspective. American Journal of Tropical Medicine and Hygiene. 1072;86(6):1072-6. doi: http://dx.doi.org/10.4269/ajtmh.2012.11-0723. PMID: 2012334646.
49. World Health Organization Regional Office for the Eastern Mediterranean. Dengue fever in Yemen. 2010;3(23). Available www.emro.who.int/surveillance-forecasting-response/weekly-epidemiological-monitor/. Accessed 17 Jan 2016.
50. Madani TA, Abuelzein ETME, Al-Bar HMS, Azhar EI, Kao M, Alshoeb HO, et al. Outbreak of viral hemorrhagic fever caused by dengue virus type 3 in Al-Mukalla, Yemen. BMC Infectious Diseases. 2013;13(1). doi: http://dx.doi.org/10.1186/1471-2334-13-136. PMID: 2013189579.
51. ProMED-mail. Dengue Fever - Yemen (10): (Shabwah). ProMED-mail. 2015; 31 Dec: 20151231.3903031. <http://www.promedmail.org>. Accessed 17 Jan 2016.
52. ProMED-mail. Dengue Fever - Yemen (05): (Shabwah) Fatal, MOH.: ProMED-mail. 5 Sept: 20150905.3625697. <http://www.promedmail.org>. Accessed 17 Jan 2016.
53. ProMED-mail. Dengue Fever - Yemen (04): (Taiz) WHO update. ProMED-mail. 29 Aug: 20150829.3608441. <http://www.promedmail.org>. Accessed 17 Jan 2016.
54. ProMED-mail. Dengue Fever - Yemen (03): Fatal, Request for Information. ProMED-mail. 18 June: 20150618.3445620. <http://www.promedmail.org>. Accessed 17 Jan 2016
