# Supplementary material for: Dengue in the Middle East and North Africa: A Systematic Review
Source: PLoS Negl Trop Dis. 2016 Dec 7;10(12):e0005194. doi: 10.1371/journal.pntd.0005194 (PMC5142774; doi:10.1371/journal.pntd.0005194)
Supplement: S3 Table — (PDF) [file pntd.0005194.s005.pdf]

S3 Table. Reported *Aedes aegypti* and *Aedes albopictus* occurrence in the Middle East and North Africa.

Country	*Aedes aegypti*	*Aedes albopictus*	Reference
**Afghanistan**	**+**	**-**	[1]
**Algeria**	***historic only***	**+**	[2, 3]
**Bahrain**	**-**	**-**	**-**
**Djibouti**	**+**	**-**	[4]
**Egypt**	**+**	**-**	[5]
**Iran**	**-**	**-**	**-**
**Iraq**	**-**	**-**	**-**
**Jordan**	**-**	**-**	**-**
**Kuwait**	**-**	**-**	**-**
**Lebanon**	**+**	**+**	[6-8]
**Libya**	***historic only***	**-**	[3]
**Morocco**	***historic only***	**-**	[3]
**Oman**	**+**	**-**	[9]
**Pakistan**	**+**	**+**	[10]
**Palestine**	**-**	**+**	[11]
**Qatar**	**-**	**-**	**-**
**Saudi Arabia**	**+**	**+**	[12-15]
**Somalia**	**+**	**-**	[16, 17]
**Sudan**	**+**	**-**	[18]
**Syria**	***historic only***	**+**	[3, 7]
**Tunisia**	***historic only***	**-**	[3]
**Turkey**	**+**	**+**	[1, 19, 20]
**U.A.E.**	**-**	**-**	**-**
**Yemen**	**+**	**-**	[21]

Historic only refers to reported occurrence of that species prior to 1960; all other reports were published on or after 1995.

U.A.E., United Arab Emirates

References

1. Kraemer MU, Sinka ME, Duda KA, Mylne AQ, Shearer FM, Barker CM, et al. The global distribution of the arbovirus vectors Aedes aegypti and Ae. albopictus. Elife. 2015;4:e08347. doi: 10.7554/eLife.08347. PMID: 26126267.
2. Izri A, Bitam I, Charrel RN. First entomological documentation of Aedes (Stegomyia) albopictus (Skuse, 1894) in Algeria. Clinical Microbiology and Infection. 2011;17(7):1116-8. doi: http://dx.doi.org/10.1111/j.1469-0691.2010.03443.x. PMID: 2011372571.
3. Holstein M. Dynamics of Aedes aegypti distribution, density and seasonal prevalence in the Mediterranean area. Bull World Health Organ. 1967;36(4):541-3. PMID: 5299448.
4. Rodier GR, Gubler DJ, Cope SE, Cropp CB, Soliman AK, Polycarpe D, et al. Epidemic dengue 2 in the city of Djibouti 1991-1992. Transactions of the Royal Society of Tropical Medicine and Hygiene. 1996;90(3):237-40. doi: http://dx.doi.org/10.1016/S0035-9203%2896%2990228-X. PMID: 1996194667.
5. Heikal OM, El-Bahnasawy MM, Morsy AT, Khalil HH. Aedes aegypti re-emerging in Egypt: a review and what should be done? Journal of the Egyptian Society of Parasitology. 2011;41(3):801-14. PMID: 22435171.
6. Knio KM, Markarian N, Kassis A, Nuwayri-Salti N. A two-year survey on mosquitoes of Lebanon. Parasite. 2005;12(3):229-35. PMID: 16218210.
7. Haddad N, Harbach RE, Chamat S, Bouharoun-Tayoun H. Presence of Aedes albopictus in Lebanon and Syria. J Am Mosq Control Assoc. 2007;23(2):226-8. doi: 10.2987/8756-971X(2007)23[226:POAAIL]2.0.CO;2. PMID: 17847859.
8. Haddad N, Mousson L, Vazeille M, Chamat S, Tayeh J, Osta MA, et al. Aedes albopictus in Lebanon, a potential risk of arboviruses outbreak. BMC Infectious Diseases. 2012;12(300). doi: http://dx.doi.org/10.1186/1471-2334-12-300. PMID: 2012729031.

9. Dengue fever. Communicable Diseases Surveillance Newsletter. September 21, 2014.
10. Jahan N. Detection of dengue viruses in aedes mosquitoes from different localities of Lahore, Pakistan. American Journal of Tropical Medicine and Hygiene. 2013;89 (5 SUPPL. 1) (pp 35). PMID: 71311948.
11. Adawi SHAA. Presence of Aedes albopictus in Palestine –West Bank. International Journal of Tropical Disease and Health. 2012;2(4):301-10.
12. Health Section of the Secretariat of the League of Nations. Dengue and dengue haemorrhagic fever. Weekly epidemiological record. 1995;70(33):237-8. PMID: 7669530.
13. Alikhan M, Al Ghamdi K, Mahyoub JA. Aedes mosquito species in western Saudi Arabia. J Insect Sci. 2014;14:69. doi: 10.1093/jis/14.1.69. PubMed PMID: 25373216.
14. Al-Azab AM, Al-Ghamdi KM, Al-Hudaib K, Zaituon AA, Shaheen MA, El-Araby W. Morphological and molecular identification of dengue fever vector Aedes aegypti (diptera: Culicidae) in Jeddah Governorate-Saudi Arabia. Journal of Pure and Applied Microbiology. 2013;7(2):1391-400. PMID: 2013521457.
15. Aziz AT, Dieng H, Ahmad AH, Mahyoub JA, Turkistani AM, Mesed H, et al. Household survey of container-breeding mosquitoes and climatic factors influencing the prevalence of Aedes aegypti (Diptera: Culicidae) in Makkah City, Saudi Arabia. Asian Pacific Journal of Tropical Biomedicine. 2012;2(11):849-57. doi: http://dx.doi.org/10.1016/S2221-1691%2812%2960242-1. PMID: 2012480605.
16. Sharp TW, Wallace MR, Hayes CG, Sanchez JL, DeFraites RF, Arthur RR, et al. Dengue fever in U.S. troops during Operation Restore Hope, Somalia, 1992- 1993. American Journal of Tropical Medicine and Hygiene. 1995;53(1):89-94. PMID: 1995228394.
17. Kyobe Bosa H, Montgomery JM, Kimuli I, Lutwama JJ. Dengue fever outbreak in Mogadishu, Somalia 2011: Co-circulation of three dengue virus serotypes. International Journal of Infectious Diseases. 2014;21:3. doi: http://dx.doi.org/10.1016/j.ijid.2014.03.412. PMID: 71633760.
18. Seidahmed OME, Hassan SA, Soghaier MA, Siam HAM, Ahmed FTA, Elkarsany MM, et al. Spatial and Temporal Patterns of Dengue Transmission along a Red Sea Coastline: A Longitudinal Entomological and Serological Survey in Port Sudan City. PLoS Neglected Tropical Diseases. 2012;6(9). doi: http://dx.doi.org/10.1371/journal.pntd.0001821. PMID: 2012579248.
19. Oter K, Gunay F, Tuzer E, Linton YM, Bellini R, Alten B. First record of Stegomyia albopicta in Turkey determined by active ovitrap surveillance and DNA barcoding. Vector Borne Zoonotic Dis. 2013;13(10):753-61. doi: 10.1089/vbz.2012.1093. PMID: 23808976.
20. Akiner MM, Demirci B, Babuadze G, Robert V, Schaffner F. Correction: Spread of the Invasive Mosquitoes Aedes aegypti and Aedes albopictus in the Black Sea Region Increases Risk of Chikungunya, Dengue, and Zika Outbreaks in Europe. PLoS neglected tropical diseases. 2016;10(5):e0004764. doi: 10.1371/journal.pntd.0004764. PMID: 27232603.
21. Fahmy NT, Klena JD, Mohamed AS, Zayed A, Villinski JT. Complete Genome Sequence of Chikungunya Virus Isolated from an Aedes aegypti Mosquito during an Outbreak in Yemen, 2011. Genome announcements. 2015;3(4). doi: 10.1128/genomeA.00789-15. PMID: 26184944.
